# Design, synthesis and anticonvulsant evaluation of novel 2,4,5-trichlorobenzenesulfonate-based dihydrothiazoles supported by *in vivo* and *in silico* studies

**DOI:** 10.1039/d6ra00305b

**Published:** 2026-03-03

**Authors:** Faiqa Noreen, Muhammad Iftikhar Shoukat, Imran Imran, Farhan Siddique, Sumaira Nadeem, Mostafa A. Ismail, Xianliang Zhao, Magdi E. A. Zaki, Sobhi M. Gomha, Zahid Shafiq

**Affiliations:** a Institute of Chemical Sciences, Bahauddin Zakariya University Multan 60800 Pakistan zahidshafiq@bzu.edu.pk; b Department of Pharmacology, Faculty of Pharmacy, Bahauddin Zakariya University Multan 60800 Pakistan; c Department of Pharmaceutical Chemistry, Faculty of Pharmacy, Bahauddin Zakariya University Multan 60800 Pakistan; d Department of Pharmacy, The Women University Multan 60000 Pakistan; e Central Labs, King Khalid University P. O. Box 960, AlQura'a Abha 61413 Saudi Arabia; f School of Biological and Chemical Engineering, Zhejiang University of Science and Technology Hangzhou 310023 Zhejiang Province China; g Department of Chemistry, Faculty of Science, Imam Mohammad Ibn Saud Islamic University (IMSIU) Riyadh 11623 Saudi Arabia; h Department of Chemistry, Faculty of Science, Islamic University of Madinah Madinah 42351 Saudi Arabia smgomha@iu.edu.sa

## Abstract

This study describes the synthesis of a new series of 2,4,5 trichlorobenzenesulfonate structural class dihydrothiazoles and assesses the potential of the compounds as antiepileptic drugs using *in vivo* methods, network pharmacology, molecular docking, and DFT computational methods. Ten compounds 3(a–j) were synthesized and characterized, and their anti-seizure effects were evaluated on the standard seizure models. *In vivo* anticonvulsant activity was explored in the 6 HZ corneal stimulation model. Compound 3c showed complete protection, with return to normal behavior in less than 10 seconds post stimulation. The rest of the compounds exhibited 25% (3a), 50% (3d), 75% (3f), and 75% (3j) protection, respectively. In the pentylenetetrazole (PTZ) chemoconvulsant model, compound 3c at 150 mg kg^−1^ showed complete protection from death and hind limb extension. None of the animals showed seizure activity for 30 minutes post-administration. In the *in silico* molecular docking, compound 3c showed the greatest anticonvulsant activity among the synthesized compounds. Compound 3c had a binding affinity of −9.7 kcal mol^−1^, while the co-crystallized reference ligand had −6.7 kcal mol^−1^ which indicates a direct correlation between binding score and experimental anticonvulsant activity. Based on both *in vivo* and *in silico* findings, compound 3c emerged as the most important candidate, demonstrating superior anti convulsant activity across multiple seizure models. These results underscore the capability of the newly synthesized 2, 4, 5-trichlorobenzenesulfonate based dihydrothiazoles as promising scaffolds for the development of new antiepileptic drugs.

## Abstract 2

A new series of 2,4,5-trichlorobenzenesulfonate-based dihydrothiazoles was synthesized and evaluated as anticonvulsant agents.

## Graphical abstract

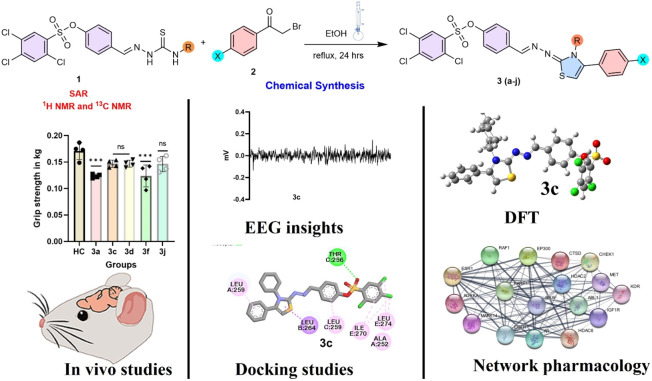

## 1. Introduction

Epilepsy is described as a chronic neurological disorder that has a direct correlation with unprovoked seizures.^1^ It possesses the capability to affect individuals in a multitude of ways relating to their socio-cultural, psychological, economic, cognitive, and neurobiological aspects.^2^ Worldwide, approximately 50 million individuals suffer from this condition, with chronic brain disorders being the most prevalent form of ailment. It is furthermore estimated that about 80 percent of afflicted individuals live in low and medium income countries, which is over 75 percent of the total estimated global figure.^3–5^ Furthermore, these individuals are suffering from a healthcare deficit in proper diagnosis and treatment. All these factors culminate in the condition being increasingly debilitating and progressive.^6^

The causes of epilepsy are complicated, since they include genetic structure, metabolism, infection, immune response, and often no identifiable cause.^7^ It is diagnosed clinically by the presence of uncontrollable and spontaneous seizures, which is abnormal electric activity of the brain 8. During the seizure, the individual may display movement, sensation, and various levels of (or no) unconsciousness.^9^ The World Health Organization states that 70 percent of epilepsy patients can be diagnosed and treated accordingly, but still with significant drawbacks.^10,11^ Antiepileptic drugs (AEDs) only control seizures in around 60–80% of patients, alongside some major drawbacks like non-selectivity, toxicity, and formulation struggles.^12^ The WC states that 30 percent of patients are still pharmaco-resistant. This demonstrates the urgent need to improve the quality of the life of patients with epilepsy around the globe.^13^ This indicates the need of developing antiepileptic drugs which are safer and more selective.

**Fig. 1:**
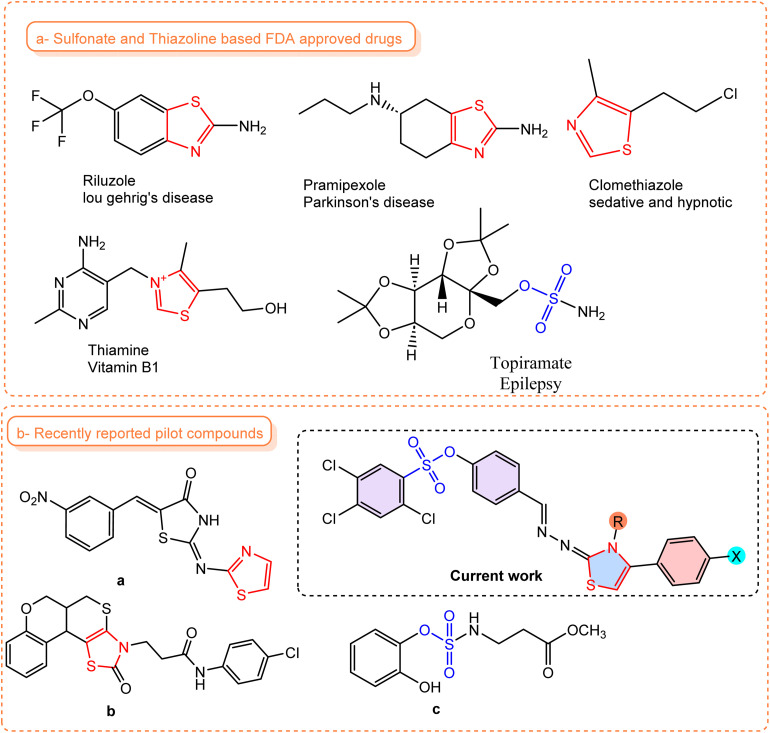
Summary of sulfonate and thiazoline-containing (a) FDA-approved drugs and (b) some recently reported sulfonate and thiazoline-based anti-epileptic drugs.

The past few years have seen sulfonate compounds focus on in medicinal chemistry prominently because of their multifunctional sulfonate derivatives antimicrobial,^14^ anticancer,^15^ anti-inflammation ^16^ and more recently, sulfonate CNS (central nervous system) activity.^17^ Inclusion of sulfonate moieties in candidate molecules and their disparity in pharmacokinetic properties, membrane permeability and solubility enables strong target interaction. Furthermore, thiazoles and dihydrothiazole heterocycles are underemphasized privileged scaffolds in drug discovery because of their CNS receptors and seizure forming and propagating enzymes.^18,19^ The strategic hybridization of sulfonate and dihydrothiazoles motifs opens up exciting avenues for designing next generation anticonvulsant agents. Such hybrid molecules assume to leverage the strengths of both structural components not only enhance anticonvulsant potency but also improve the pharmacological profile and reduce off-target effects. Some of the pilot compounds having sulfonate or thiazoline have been depicted in Fig. 1b.^20–22^

In this regard, we investigated the design, synthesis and extensive characterization of a novel series of potential antiepileptic dihydrothiazoles 3(a–j) derived from 2,4,5-trichlorobenzenesulfonate, employing a combination of *in vivo*, *in silico* approaches, and network pharmacology approaches.

To better un-veil the molecular mechanisms behind the investigated anticonvulsant action, a reliable computational approach was planned to support the experimental findings. Molecular docking evaluated the synthesized drug's binding interactive modes with the GABA-A receptor,^23^ which is a crucial target in the treatment plan of epilepsy. The DFT was incorporated to explore the electrical features of these synthesized series of compounds.^24^ Network pharmacology approach was employed to identify possible receptor targets and associated signaling pathways that these compounds influence, to explain their multi-target therapeutic ability.^25^ Thus, integrated computational techniques supported the experimental outcomes. We aim to construct a novel class of antiepileptic drugs with enhanced efficacy and reduced toxicity by utilizing the complementary pharmacophoric features of the sulfonate and dihydrothiazole scaffolds like favorable membrane permeability, hydrogen bond donors and acceptors, and electron-rich domains. The proposed research attempts to improve the current situation of epilepsy patients with the greatest unmet clinical needs, especially those with drug resistant forms of the disorder. Moreover, it aims to facilitate the continuous evolution of epilepsy management by providing new effective antiepileptic drugs.

## 2. Results and discussion

### 2.1. Chemistry

**Scheme 1:**
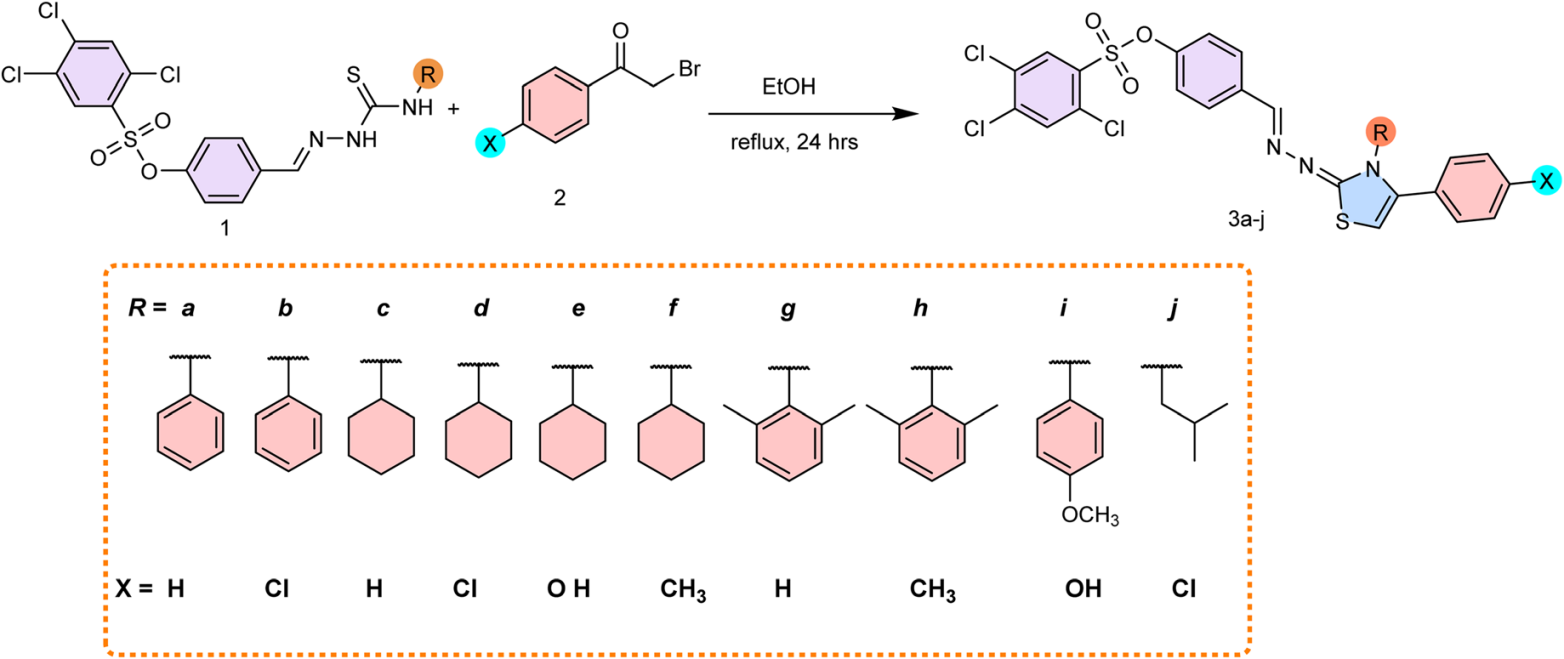
Synthesis of aryl sulfonate-based dihydrothiazoles.

A novel series of 2,4,5-trichlorobenzenesulfonate based dihydrothiazoles 3(a–j) was synthesized as depicted in Scheme 1. The synthesis involved the cyclocondensation of sulfonate thiosemicarbazone intermediate 1(a–j) with substituted phenacyl bromide (2) in ethanol under reflux for 24 hours, yielding the target dihydrothiazole derivatives 3(a–j). The structures of synthesized compounds were confirmed using various advanced spectroscopic methods. In the ^1^H NMR spectra of the 2,4,5-trichlorobenzenesulfonate based dihydrothiazoles 3(a–j), a prominent singlet was observed in the chemical shift range of *δ* 8.36–8.34 ppm, which is attribute to the imine (CH=N) protons, confirming the presence of thiosemicarbazone linkage in the core structure. For compounds containing OH proton their singlet come in the range of *δ* 9.84–9.63 ppm, reflecting the strong deshielding effect of adjacent electron withdrawing substituents. Aromatic protons located between two C–Cl carbons on the trichloro phenyl ring resonated between *δ* 8.10–8.07 ppm, while proton to the *ortho* of sulfonyl group on trichloro phenyl ring resonated between *δ* 8.10–8.28 ppm. The thiazole ring proton was observed as singlet in the *δ* 6.76–6.32 ppm region. In the ^13^C NMR spectrum, the signal observed in the range of *δ* 169–171 ppm is attributed to C–O carbon adjacent to sulfonyl oxygen, which is significantly deshielded due to the strong electron withdrawing effect of sulfonate group. The signal observed in the range of *δ* 149–150 ppm is assigned to C=N carbon of thiosemicarbazone moiety, reflecting the characteristic chemical shift of imine carbon.

### 2.2. 6 Hz seizure model

**Fig. 2:**
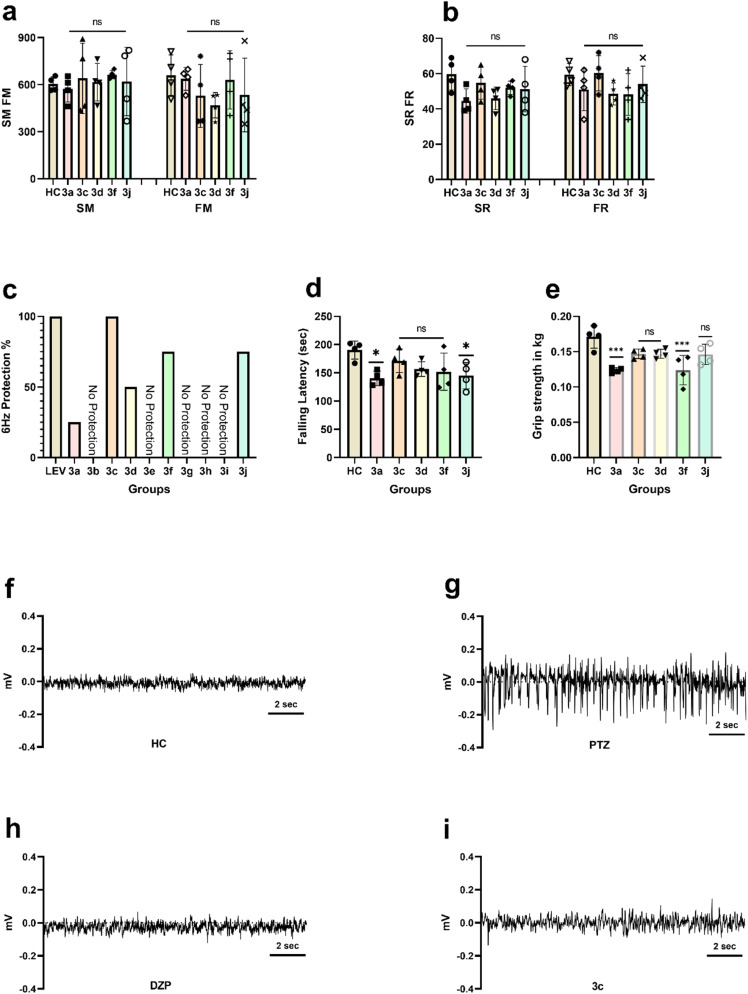
Results of mice (*n* = 4) of different treatment groups were represented after applying one way ANOVA multiple comparison followed by *post hoc* Dunnett test and descriptive statistics. Significant differences were represented by * = *P* < 0.05, ** = *P* < 0.01, *** = *P* < 0.001 and **** = *P* < 0.0001. Locomotor activity test representing (a) horizontal movements in terms of slow movements and fast movements while (b) vertical movements in terms of slow rearing and fast rearing. (c) Mice percentage protection of different groups in 6 Hz seizure model. (d) Motor co-ordination test showing falling latency of mice from rod of treadmill rotarod. (e) Mice forelimb muscle grip strength represented in kg. 10 s electrogram seizures magnification represented in mV of (f) baseline EEG of healthy mice, (g) acute 80 mg per kg PTZ group, (h) Diazepam 5 mg kg^−1^, and (i) EEG of 3c compound treated mice.

All the animals in group 3c returned to their normal behavior within 10 s post 6 Hz corneal stimulation and showed complete protection while compounds 3a, 3d, 3f and 3j showed 25%, 50%, 75% and 75% protection respectively. The results were compared with LEV group that showed complete protection from psychomotor seizure progression in this 6 Hz seizure model as given in Fig. 2.

### 2.3. PTZ seizure model

3c compound tested in chemoconvulsant PTZ seizure model manifested complete protection from death and even hind limb extension as all the animals in this group did not show hind limb extension in 30 minutes of post PTZ observation at dose 150 mg kg^−1^. At dose 100 mg kg^−1^3c compound showed 75% protection while showing significant delay in hind limb extension. There was 25% protection from death in groups 3f and 3j at both doses but there was no significant difference in latency to hind limb extension and death compared to PTZ group. Compounds 3c and 3d exhibited significant difference compared to PTZ group in latencies to myoclonic seizure tonic–clonic seizure especially at higher dose 150 mg kg^−1^. 3d compound exhibited significant delay in myoclonic and tonic–clonic seizures and even in latency to hind limb extension at both doses. 25% protection is observed at 150 mg kg^−1^ and notable delay in death latency of the remaining animals is observed in 3c group. The detailed results are displayed in Table 1.

**Table 1:** Table showing results of synthetic compounds tested in chemoconvulsant PTZ seizure model with mean ± SD, **** = *P* < 0.0001. One way ANOVA *post hoc* Dunnett test and descriptive statistics were applied while analyzing and results of synthetic compounds were compared with PTZ group (*n* = 4)

Group	Dose (mg kg^−1^)	Latency to myoclonic seizure (s)	Latency to tonic–clonic seizure (s)	Latency to hind limb extension (s)	Latency to death (s)	Percent protection
PTZ	80	79.5 ± 22.88	111.5 ± 56.97	121.5 ± 56.85	163.8 ± 48.54	0%
DZP	5	783.5 ± 33.0****	1800 ± 00****	1800 ± 00****	1800 ± 00****	100%
3a	100	102.5 ± 15.55	157.5 ± 11.9	187.3 ± 37.82	180.5 ± 14.64	0%
3b	100	121.3 ± 18.28	178.8 ± 17.5	189.5 ± 21.75	218.8 ± 12.84	0%
3c	100	118.8 ± 13.77	770.3 ± 247.0	1411 ± 461.70****	1651 ± 297.50***	75%
	150	176.5 ± 36.52****	1449 ± 180.7****	1800 ± 0****	1800 ± 0****	100%
3d	100	133.0 ± 6.272**	690.8 ± 342.9***	736.3 ± 368.5***	767.5 ± 378.6	0%
	150	140.3 ± 6.652**	581.8 ± 252.3**	643.3 ± 243.0**	986.3 ± 583.1	25%
3e	100	137.3 ± 13.05**	173.0 ± 9.274	206.5 ± 40.32	231.3 ± 46.61	0%
3f	100	117.8 ± 12.12	356.3 ± 269.5	396 ± 316.2	830 ± 706.8	25%
	150	133.3 ± 15.06**	383 ± 259.4	503.5 ± 248.3	882.2 ± 672.4	25%
3g	100	135.3 ± 27.66**	176.8 ± 64.52	205.3 ± 71.44	221.8 ± 74.96	0%
3h	100	118.8 ± 21.7	163.5 ± 35.2	177.5 ± 41.51	190 ± 43.01	0%
3i	100	107.5 ± 11.68	146.0 ± 62.71	188.3 ± 88.97	244.8 ± 165.7	0%
3j	100	125.5 ± 12.23*	163.3 ± 21.5	199 ± 50.64	611.3 ± 792.9	25%
	150	153.5 ± 38****	222 ± 88.88	261 ± 92.47	699.3 ± 738.6	25%

### 2.4. EEG acquisition

Baseline EEG of mice with implanted cortical electrodes was recorded and mice were given 3c 150 mg per kg compound. After half an hour mice were injected with PTZ 80 mg kg^−1^ and vEEG recording continued for 30 min while one group was injected with PTZ only to observe electrographic seizures as shown in Fig. 2. High amplitude and increased frequency in EEG of PTZ treated mice was observed compared to 3c treated mice as shown in electrogram magnification of baseline, 3c and PTZ treated mice in Fig. 2. Less electrographic seizures were observed in 3c compared to acute PTZ control for 10 s activity after first epileptic spike in recorded EEG.

### 2.5. Locomotor activity test

Locomotor activity of mice treated with compounds showing some protection in seizure models at maximum doses, was compared with healthy mice. No significant difference in horizontal (SM and FM) and vertical (SR and FR) locomotor activity was observed with ANOVA applied *P* values 0.8026 and 0.2322 respectively. Comparison of synthetic compounds treated groups with healthy group showed no significant difference in locomotor activities as all of the individual comparison *P* values are greater than 0.05, demonstrating that these compounds crossed blood–brain barrier and suppressed epileptic discharges somehow while not affecting psychomotor activities of mice as shown in Fig. 2.

### 2.6. Grip strength test

Forelimb grip strength of compounds treated mice in kg was compared with healthy mice. Mice treated with 3c, 3d and 3j compounds represented no significant difference in forelimb muscle grip strengths compared to healthy mice as their *P* values are 0.0634, 0.0651 and 0.0548 respectively while 3a and 3f compounds manifested significant decline in forelimb muscle grip strengths with *P* = 0.0003 for both as represented in Fig. 2.

### 2.7. Motor co-ordination test

There was no significant difference in muscle co-ordination of mice treated with compounds 3c, 3d and 3f compared to healthy mice as *P* values were 0.5869, 0.1272 and 0.0716 respectively. While compounds 3a and 3j resulted in markedly decrease in muscle coordination as their *P* values were 0.014 and 0.0276. Mice treated with compounds 3a and 3j significantly declined their falling latency compared to healthy mice. Mice treated with 3c, 3d and 3f compounds tried to cling to the rotating rod of treadmill rotarod like healthy mice and their falling latency did not show any significant difference representing well muscle co-ordination as represented in Fig. 2.

### 2.8. Structure activity relationship (SAR)

A systematic evaluation of the ten synthesized derivatives in the 6 Hz psychomotor and PTZ-induced seizure models revealed that anticonvulsant activity is governed by the substituent at the thiazole N-3 position and X substituent on phenyl ring. When N-3 bears an aromatic phenyl ring (compound 3a) or (compound 3b), activity is completely absent 3a: 0% protection at 100 mg kg^−1^ and 3b: 0% protection in PTZ and 6 Hz at 100 mg kg^−1^, with no significant effect on seizure latencies. In striking contrast, all compounds carrying a cyclohexyl group at N-3 (3c, 3d, 3f) displayed markedly improved profiles, demonstrating that a lipophilic, saturated alicyclic substituent at this position is essential for meaningful anticonvulsant potency.

**Fig. 3:**
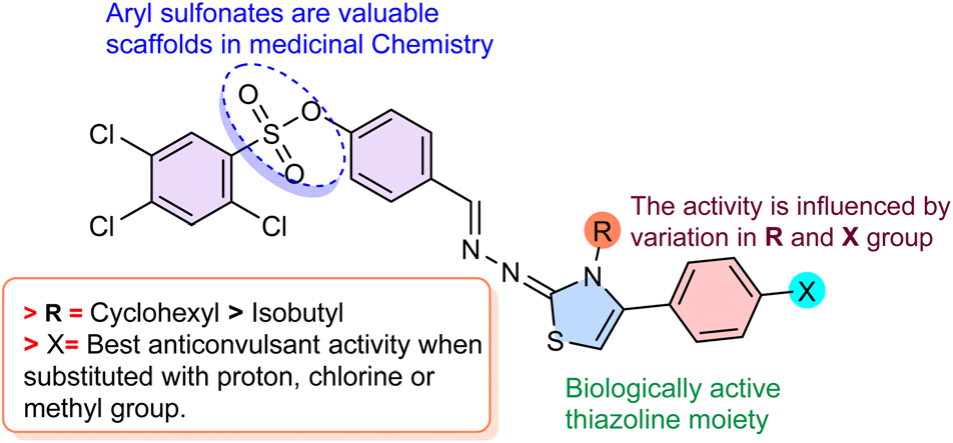
Structure–activity relationship (SAR) of the synthesized compounds.

Among the *N*-cyclohexyl analogues, compound 3c (C-4 = unsubstituted phenyl) emerged as the standout derivative, providing complete protection in the 6 Hz model at both 100 and 150 mg kg^−1^ (identical to levetiracetam) and full protection against hind-limb extension and mortality in the PTZ model at 150 mg kg^−1^, with 75% protection and highly significant prolongation of all seizure phases at 100 mg kg^−1^. The unsubstituted phenyl in 3c provides a neutral, hydrophobic aryl domain that likely supports pi–pi stacking or van der Waals interactions with aromatic residues in binding sites, achieving full protection. Substitution on the C-4 phenyl ring with either 4-chloro (3d) or 4-methyl (3f) groups substantially diminished activity: 3d retained moderate effects in the PTZ model (significant delays in myoclonic, tonic–clonic and hind-limb extension latencies; 25–50% protection from death) but was completely inactive in the 6 Hz model, while 3f showed only marginal 25% protection in PTZ and none in 6 Hz. These findings establish a very narrow SAR window in which optimal broad-spectrum anticonvulsant activity requires the precise combination of N-3 cyclohexyl and C-4 unsubstituted phenyl substitution. Compounds 3g and 3h, bearing *R* = 2,6-dimethyl phenyl, displayed complete inactivity (0% protection in both 6 Hz and PTZ models), confirming that introduction of an aromatic ring at N-3, is detrimental due to excessive π-character and/or metabolic liability. Similarly, 3i (*R* = 4- methoxy phenyl) yielded zero protection, highlighting that polar, hydrogen-bond-donating substituents at N-3 dramatically reduce lipophilicity and prevent blood–brain barrier penetration. Similarly, the isobutyl group in 3j, while aliphatic, is less bulky and hydrophobic, offering insufficient membrane permeability or target affinity, yielding 25% protection in PTZ, and 75% in 6 Hz at 150 mg kg^−1^ (Fig. 3).

### 2.9. Molecular docking studies

The molecular docking studies shed light on remarkable deeper insights related to the anticonvulsant activity of the studied series of compounds where experimentally hit compound 3c exhibited the strong binding affinity (−9.7 kcal mol^−1^), owing to the natively adhered co-crystallized ligand (−6.7 kcal mol^−1^). Its greater binding score shows correlation with its notable experimental anticonvulsant activity. The *in silico* studies also discovered that compound 3g is found to be the top leading one, carrying the highest binding affinity score of −10.2 kcal mol^−1^.

**Fig. 4:**
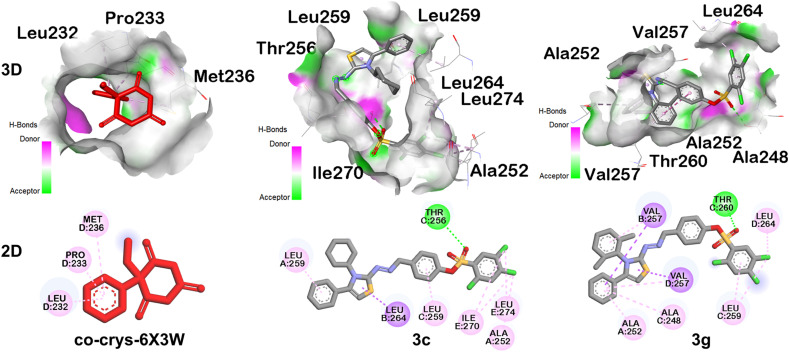
3D interactive view having H-bond surface and 2D interaction of co-crystallized ligand, 3c, and 3g showing key amino acid residues for anticonvulsant activity.

A comprehensive analysis of their binding interactions (Table 2 and Fig. 4) reflected that all the compounds of series scored considerably better than the co-crystallized ligand (−6.7 kcal mol^−1^). It suggests strong forecasted binding ability in the chosen binding site(s), which is highly consistent with the experimentally observed anticonvulsant activity for at least 3c. As far as the binding mode and interaction analysis of 3c was concerned against the co-crystallized ligand, it established the H-bonding in chain C with amino acid residue THR256 at the corresponding distance of 2.88 Å. In addition, multiple hydrophobic interactions with residues LEU264 (chain-B), ILE270 (chain-E), LEU274 (chain-E), ALA252 (chain-A), LEU259 (chains-C/A) were seen along with various pi–sigma, alkyl/pi–alkyl interactions. The compound 3g exhibited the H-bond formation *via* chain-C through amino acid residue THR260 at a relevant distance of 2.66 Å. It incorporated various hydrophobic liaisons *via* the amino acids VAL257 (chains-B/D), LEU259 (chain-C), LEU264 (chain-D), ALA252 (chain-A), ALA248 (chain-C), *etc.* were noted along with added pi-type interactions (pi–sigma, pi–pi T-shaped, pi–alkyl). On the contrary, the co-crystallized ligand of our target protein (6X3W) has a limited portfolio in this regard, showing hydrophobic contacts with LEU232, PRO233, MET236, all observed in chain-D at varying distances of ∼4.4–5.1 Å. Supplement to it, the native ligand reported none of the H-bond interactive connections compared to 3c and 3g. 3c is experimentally active (hit) and docking score (−9.7) is favorable which is attributable with consistent and plausible binding mode to the GABA A site(s). 3g showed even better predicted affinity (−10.2) and may be a promising *in silico* lead.

Molecular docking results carrying the docking scores (kcal mol^−1^) of 3a–3j, as well as various interactions observed in hit compounds (3c and 3g) against target protein (co-crystallized ligand)

**Table:** 

Docking scores of all investigated compounds with the target protein (PDB ID 6X3W)					
Code	Docking score (kcal mol^−1^)	Code	Docking score (kcal mol^−1^)	Code	Docking score (kcal mol^−1^)
3a	−9.2	3e	−9.3	3i	−8.7
3b	−9.7	3f	−9.6	3j	−8.2
3c	−9.7	3g	−10.2	**Co-crystallized ligand**	−6.7
3d	−9.6	3h	−10.0

**Table:** 

Molecular docking interactions data of experimentally hit (3c) and docking hit (3g) compounds				
Code	Key amino acid residues	Distance measured in Å	Interaction	Type of interaction
3c	C: THR256	2.88	Hydrogen bond	Conventional hydrogen bond
	B: LEU264	3.82	Hydrophobic	Pi–sigma
	E: ILE270	4.67	Hydrophobic	Alkyl
	E: LEU274	4.64	Hydrophobic	Alkyl
	A: ALA252	3.40	Hydrophobic	Alkyl
	E: ILE270	4.77	Hydrophobic	Alkyl
	C: LEU259	4.51	Hydrophobic	Pi–alkyl
	A: LEU259	4.88	Hydrophobic	Pi–alkyl
3g	C: THR260	2.65	Hydrogen bond	Conventional hydrogen bond
	B: VAL257	3.98	Hydrophobic	Pi–sigma
	D: VAL257	3.71	Hydrophobic	Pi–pi T-shaped
	C: LEU259	4.15	Hydrophobic	Alkyl
	D: LEU264	5.07	Hydrophobic	Alkyl
	A: ALA252	5.24	Hydrophobic	Pi–alkyl
	C: ALA248	4.94	Hydrophobic	Pi–alkyl
	D: VAL257	4.48	Hydrophobic	Pi–alkyl
	B: VAL257	5.01	Hydrophobic	Pi–alkyl
	C: LEU259		Hydrophobic	Pi–alkyl
Co-crys-6X3W	D: LEU232	4.44	Hydrophobic	Pi–alkyl
	D: PRO233	4.64	Hydrophobic	Pi–alkyl
	D: MET236	5.14	Hydrophobic	Pi–alkyl

### 2.10. Drug-likeness and BBB prediction

Drug-likeness scores and BBB permeability values for series of compounds 3(a–j) were obtained using MolSoft software as tabulated in Table 3. Drug-likeness scores ranged from −0.17 to 1.00, where positive values (*e.g.*, 3c–3j except 3g and 3h) suggest structural features consistent with approved drugs, while negative values (3a, 3g, 3h) indicate lower drug-like character. BBB values (1.83–3.77) were uniformly classified as moderately permeable, suggesting that all compounds are predicted to cross the BBB to some extent but are not highly permeable. Among all the compounds, 3f exhibited the highest predicted BBB value (3.77), whereas compound 3i exhibited the lowest (1.83). Compound 3j demonstrated the highest overall drug-likeness score (1.00).

**Table 3:** The blood–brain barrier score of studied compounds, BBB score: 6 shows high, 0 shows low (DOI: https://10.1021/acs.jmedchem.9b01220)

Compound	Drug likeness score	BBB value	BBB interpretation
3a	−0.09	2.89	Moderately permeable
3b	0.06	3.22	Moderately permeable
3c	0.71	3.41	Moderately permeable
3d	0.99	3.40	Moderately permeable
3e	0.94	2.69	Moderately permeable
3f	0.77	3.77	Moderately permeable
3g	−0.17	2.88	Moderately permeable
3h	−0.16	2.86	Moderately permeable
3i	0.03	1.83	Moderately permeable
3j	1.00	3.43	Moderately permeable

### 2.11. DFT calculations

#### 2.11.1. Geometry optimization and energetic parameters

**Fig. 5:**
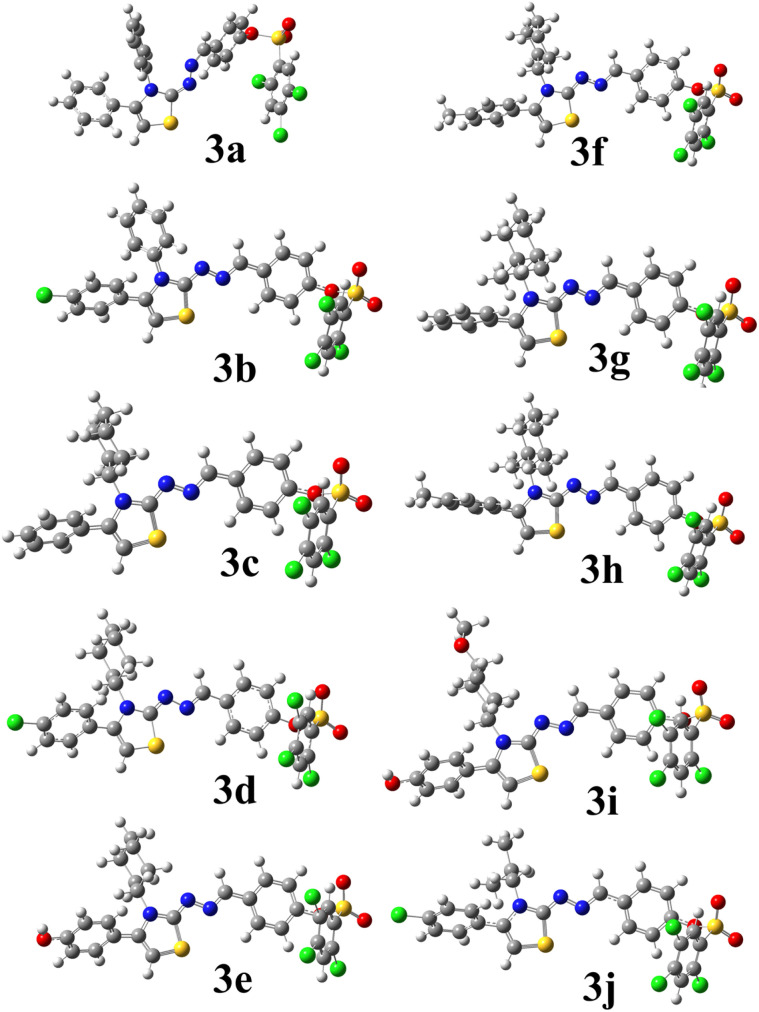
Optimized structures of the investigated compounds at DFT/B3LYP/6-311G(d,p)/GD3 calculations in the gas phase.

The optimized geometries of the compounds' series 3(a–j) were obtained at the B3LYP/6-311G(d,p)/GD3 level of theory in the ground (S_0_) state (Fig. 5). The corresponding total electronic energies, dipole moments, polarizabilities, and frontier molecular orbital (FMO) energies are summarized in Table 4.

**Table 4:** Energetic parameters of the series of compounds using DFT/B3LYP/6-311G(d,p)/GD3 in the S_0_ gas phase

Compound	Optimization energy (a.u.)	Dipole moment (debye)	Polarizability (a.u.)	HOMO (eV)	LUMO (eV)	HOMO–LUMO (Δ*E*, eV)
3a	−3645.15	8.29	463.09	−5.44	−2.20	3.25
3b	−4104.79	6.70	505.30	−5.55	−2.26	3.29
3c	−3648.81	9.32	484.36	−5.36	−2.20	3.16
3d	−4108.43	7.14	502.71	−5.46	−2.24	3.22
3e	−3724.06	9.60	491.48	−5.32	−2.20	3.13
3f	−3688.14	10.05	501.33	−5.32	−2.19	3.13
3g	−3727.47	9.22	503.44	−5.36	−2.20	3.16
3h	−3766.80	9.97	519.63	−5.32	−2.19	3.13
3i	−3838.62	8.48	508.43	−5.37	−2.22	3.16
3j	−4030.99	7.10	482.91	−5.50	−2.24	3.26

The optimization energies ranged from −3645.15 a.u. (3a) to −4108.43 a.u. (3d), indicating well-stabilized structures after geometry convergence. The calculated dipole moments varied between 6.70 D (3b) and 10.05 D (3f), reflecting different degrees of charge separation across the molecular framework due to the nature and position of substituents. Compounds 3e to 3h exhibited relatively higher dipole moments (>9 D), suggesting greater molecular polarity and potential for stronger intermolecular interactions in polar environments.

The polarizability (〈*α*〉) values ranged from 463.09 to 519.63 a.u., revealed that the electron cloud distortion increases with the introduction of electron-donating substituents. Compound 3h possessed the highest polarizability (519.63 a.u.), consistent with its high dipole moment, implying enhanced charge delocalization and improved electronic response under an external field.

#### 2.11.2. Frontier molecular orbital (FMO) analysis

**Fig. 6:**
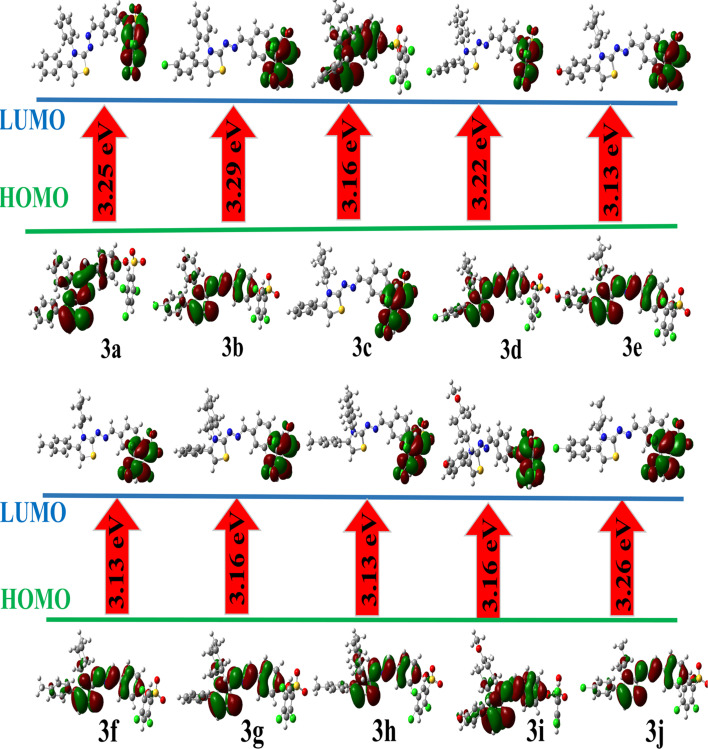
HOMO–LUMO contour plots of investigated series of compounds at DFT/B3LYP/6-311g(d,p)/GD3 calculations in the gas phase.

The FMO energies, including HOMO, LUMO, and their energy gap (Δ*E* = *E*_LUMO − *E*_HOMO) (Fig. 6), provide insight into the electronic transitions, reactivity, and stability of the molecules. The HOMO energies ranged from −5.55 eV (3b) to −5.32 eV (3e, 3f, 3h), whereas LUMO energies were found between −2.26 eV (3b) and −2.19 eV (3f, 3h). The computed HOMO–LUMO energy gaps (Δ*E*) spanned from 3.13 to 3.29 eV.

A smaller Δ*E* indicates higher electronic delocalization and greater chemical reactivity. Compounds 3e, 3f, and 3h exhibited the lowest Δ*E* (3.13 eV), implying higher softness and potential ease of charge transfer, which may correlate with better biological or photophysical activity. Conversely, compound 3b displayed the largest Δ*E* (3.29 eV), suggesting enhanced kinetic stability but lower reactivity.

#### 2.11.3. Global quantum chemical descriptors

The global reactivity descriptors including the chemical potential (*µ*), electronegativity (*χ*), chemical hardness (*η*), softness (*ζ*), electrophilicity index (*ω*), ionization potential (*I*), and electron affinity (*A*) were computed based on HOMO–LUMO energies (Table 5). These parameters elucidate the inherent stability and reactivity trends among the series.

**Table 5:** The Quantum chemical descriptors of the series of compounds using DFT/B3LYP/6-311g(d,p)/GD3 in the S_0_ gas phase

Compound	Chemical potential *µ* (eV)	Electro-negativity *χ* (eV)	Chemical hardness *η* (eV)	Chemical softness *ζ* (eV)	Electrophilicity index (eV)	Ionization potential *I* (eV)	Electron affinity *A* (eV)
3a	−3.82	3.82	1.62	0.31	4.49	5.44	2.20
3b	−3.90	3.90	1.64	0.30	4.64	5.55	2.26
3c	−3.78	3.78	1.58	0.32	4.53	5.36	2.20
3d	−3.85	3.85	1.61	0.31	4.60	5.46	2.24
3e	−3.76	3.76	1.56	0.32	4.52	5.32	2.20
3f	−3.75	3.75	1.56	0.32	4.50	5.32	2.19
3g	−3.78	3.78	1.58	0.32	4.53	5.36	2.20
3h	−3.76	3.76	1.57	0.32	4.50	5.32	2.19
3i	−3.79	3.79	1.58	0.32	4.56	5.37	2.22
3j	−3.87	3.87	1.63	0.31	4.60	5.50	2.24

The chemical potential (*µ*) values ranged from −3.90 to −3.75 eV, indicating comparable tendencies to exchange electrons with the environment. The corresponding electronegativity (*χ*) followed the same trend, signifying similar electron-accepting abilities. The chemical hardness (*η*) varied slightly between 1.56–1.64 eV, while chemical softness (*ζ*) ranged from 0.30–0.32 eV^−1^, confirming that compounds 3e–3h are relatively softer and more reactive.

The electrophilicity index (*ω*) spanned from 4.49 to 4.64 eV, denoting moderate electrophilic character. Compound 3b exhibited the highest *ω* (4.64 eV), implying a stronger tendency to accept electrons during interactions. Moreover, the ionization potential (*I*) and electron affinity (*A*) values further supported the FMO analysis, as compounds with higher *I* (*e.g.*, 3b) and lower *A* showed reduced reactivity.

#### 2.11.4. Structure–property correlations

The combined analysis of FMO and quantum descriptors reveals a clear substituent effect on electronic properties. Molecules bearing electron-donating groups (such as 3e–3h) displayed lower Δ*E*, higher dipole moments, and greater polarizability, indicating enhanced intramolecular charge transfer and potential for stronger intermolecular interactions. On the other hand, electron-withdrawing substituents (*e.g.*, in 3a, 3b, 3d) contributed to increased hardness and stability but lower reactivity. Overall, compounds 3e, 3f, and 3h emerged as the most electronically responsive and chemically active members of the series, while 3b represented the most stable yet least reactive molecule.

#### 2.11.5. MEP interpretation

**Fig. 7:**
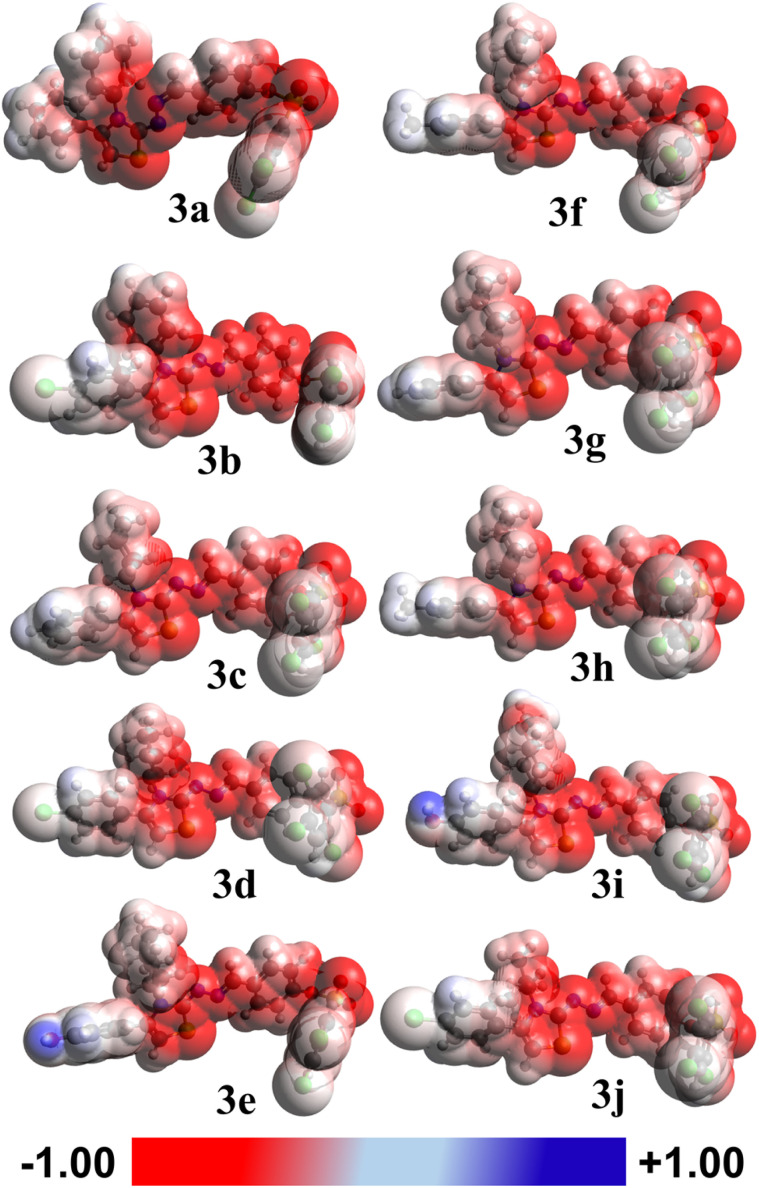
MEP maps of 3(a–j) computed at the B3LYP/6-311G(d,p)/GD3 level in the gas phase.

The MEP maps for compounds 3a–3j, calculated at the B3LYP/6-311G(d,p)/GD3 level of theory, are presented in Fig. 7. The color-coded surfaces represent the distribution of electrostatic potential around the electron density of each molecule, providing interesting insights into the charge distribution and possible sites for electrophilic and nucleophilic attack.

The color scale ranges from −1.00 (red) to +1.00 (blue) a.u., where red regions indicate areas of negative potential (electron-rich sites), often associated with electronegative atoms such as oxygen or nitrogen and are promising for electrophilic attack. Blue regions denote positive potential (electron-deficient areas), typically located near hydrogen atoms attached to electron-withdrawing groups, and are favorable for nucleophilic attack. White/light regions correspond to neutral or weakly polarized zones of the molecular surface.

Across all compounds, strong red zones were evident around carbonyl (C=O) oxygen atoms and aromatic heteroatoms, confirming these as the most electron-rich and reactive sites toward electrophiles. Conversely, blue regions appeared near hydrogen atoms attached to nitrogen or oxygen, indicating electron-poor regions prone to nucleophilic interactions.

Among the studied molecules, 3e, 3f, and 3h exhibited more intense red and blue contrasts, suggesting enhanced charge separation and stronger local dipoles. This is in excellent agreement with their higher calculated dipole moments and polarizabilities (Table 4). The vivid charge contrast implies an increased potential for intermolecular charge transfer, consistent with their lower HOMO–LUMO energy gaps (3.13 eV).

The MEP distributions therefore confirm that electronic substituents significantly influence the molecular reactivity and stability. Electron-donating groups increase electron density on aromatic frameworks, enhancing nucleophilicity, while electron-withdrawing substituents promote electrophilic centers and stabilize the charge distribution. These features collectively rationalize the observed reactivity trends and quantum chemical descriptors derived from DFT.

### 2.12. Network pharmacology

#### 2.12.1. Protein–protein interactions and compound-target-pathway network

**Fig. 8:**
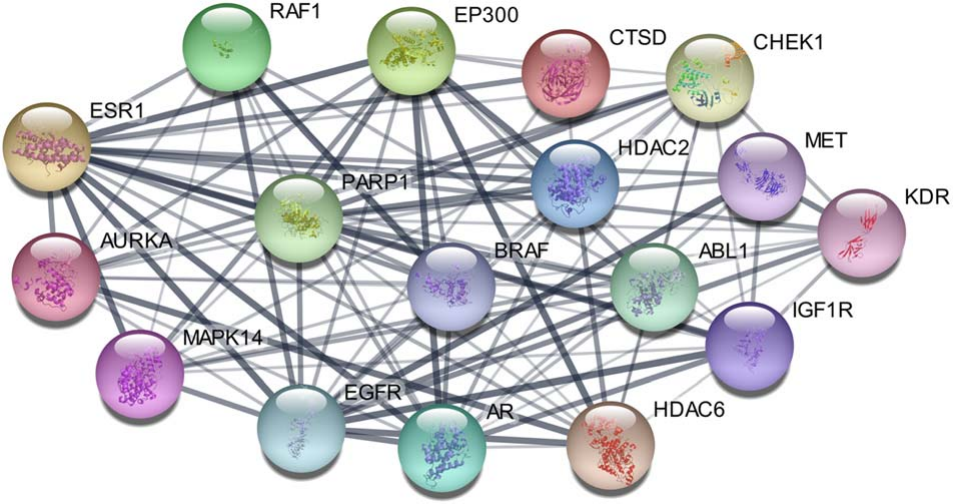
Protein interaction network generated from the STRING database.

STRING analysis identified EGFR, BRAF, RAF1, PARP1, and ESR1 as central hubs in protein–protein interaction (PPI) network indicating their key role in protein interaction dynamics, as illustrated in Fig. 8. The denser inter-connective pattern persuaded that these targets play their interactive roles in overlapping biological processes and associated potentially mediating synergistic effects of the series of studied compounds.

**Fig. 9:**
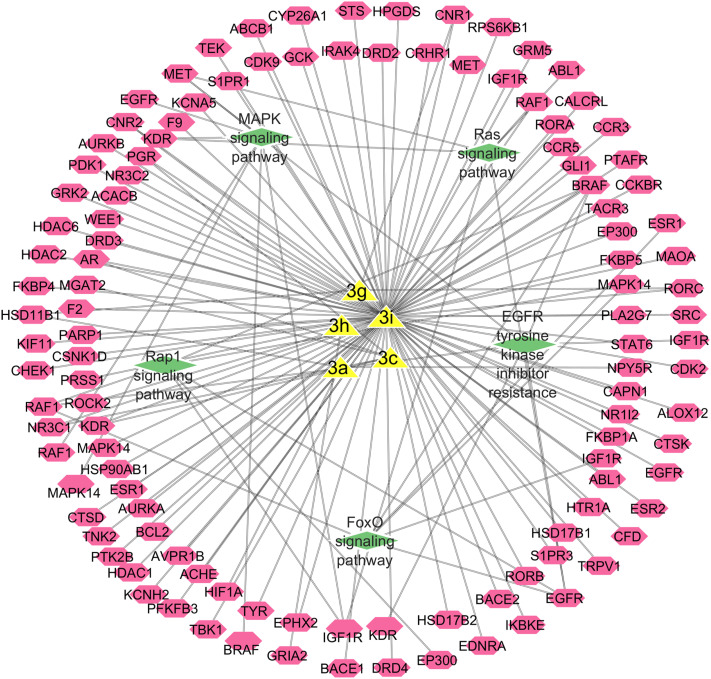
Network pharmacology map for investigated compounds (yellow) showing their interactions with predicted targets (pink) and associated signaling pathways (green).

Fig. 9 showed compound-target-pathway network, where yellow colored triangles represented the investigated compounds. Pink nodes showed the targets such as EGFR, IGF1R, ABL1, MAPK14, HDAC6, PARP1 *etc.* Green labels represent major signaling pathways (MAPK signaling pathway, Rap1 signaling pathway, EGFR tyrosine kinase inhibitor resistance, and FoxO signaling pathway). This network highlights multi-target interactions, suggesting potential for polypharmacology and pathway modulation. These compounds exhibited multi-target and multi-pathway mechanisms, a characteristic of network pharmacology-based drug design. Central hubs (*e.g.*, EGFR and BRAF) may serve as key mediators of biological effects, providing targets for experimental validation, where crosstalk between MAPK and Rap1 pathways could amplify or regulate therapeutic outcomes. This network suggests potential combinatorial effects, demanding further *in vitro* and *in vivo* studies. Network pharmacology analysis reveals that the investigated compounds act on multiple protein targets connected through critical signaling pathways, supporting their potential as multi-functional therapeutic agents. Future work should validate these interactions experimentally and evaluate their biological outcomes in disease models.

#### 2.12.2. Gene-ontology (GO) enrichment analysis

**Fig. 10:**
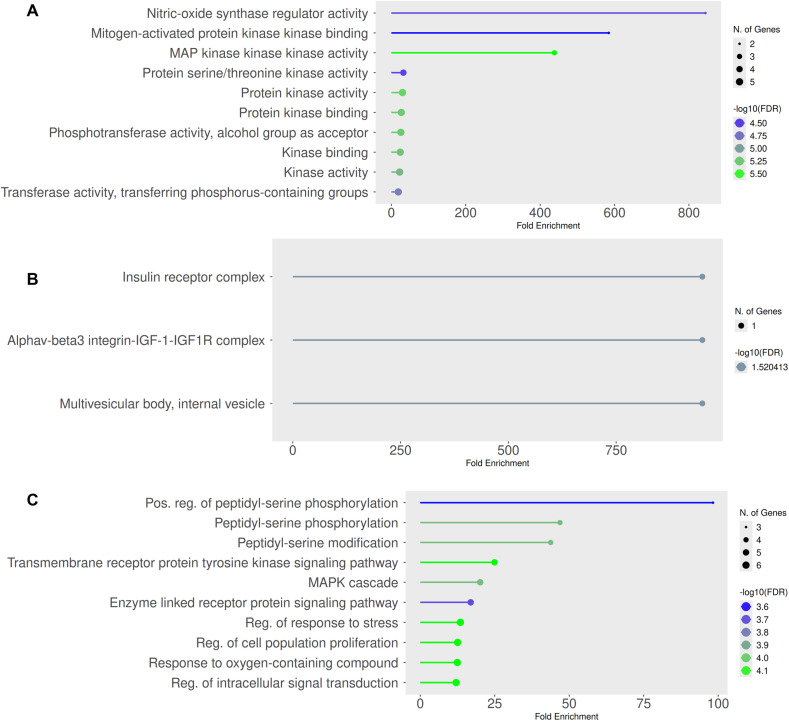
GO-enrichment analysis. Biological processes (panel-A), cellular function (panel-B), and molecular processes (panel-C).

The three components of GO-enrichment analysis was performed to visualize the plots of molecular functions (MF), cellular components (CC), and biological processes (BP), as shown in Fig. 10. The enrichment analysis of molecular functions revealed pronounced involvement of iron-associated activities. Ferric iron binding showed one of the highest enrichments, with a fold increase of about 350, supported by eight genes with −log_10_(FDR) ∼ 2.2, pointing to disrupted iron metabolism in epileptic pathology. Similarly, ferrous iron binding was enriched nearly 50-fold, represented by four genes, highlighting the role of redox imbalance and ferroptosis in neuronal vulnerability. Another notable category was metallo-endopeptidase activity, enriched by approximately 60-fold with five contributing genes and a −log_10_(FDR) of 2.2, suggesting dysregulation of neuropeptide processing that can alter seizure thresholds.

At the level of cellular component, vesicle-associated compartments were found to be emerged prominently. Secretory vesicles displayed nearly nine-fold enrichment, supported by ten genes and a −log_10_(FDR) value close to 3.0, underscoring the significance of vesicle-mediated neurotransmitter release in seizure dynamics. Cytoplasmic vesicle lumen also appeared enriched about eight-fold, with a −log_10_(FDR) around 2.0, emphasizing the contribution of vesicular trafficking to synaptic balance. In addition, extracellular exosomes showed −log_10_(FDR) of 2.5, aligning with increasing evidence that exosomal signaling contributes to neuro-inflammation and offers potential biomarker sources in epilepsy.^26^

Among biological processes, several categories central to neuronal excitability are enriched. The response to peptide hormone process exhibited roughly twelve-fold enrichment, with twelve genes and a −log_10_(FDR) of 4.0, consistent with modulatory effects of neuropeptides such as neuropeptide Y (NPY) and ghrelin on seizure susceptibility. The cellular response to nitrogen compound category was also strongly enriched, about eleven-fold with twelve genes and a −log_10_(FDR) of 4.0, pointing to the pathological role of dysregulated glutamate and GABA signaling in excitotoxicity. In addition, cellular homeostasis depicted a six-fold enrichment supported by ten genes with a −log_10_(FDR) near 3.5, highlighting that how impaired ionic and calcium balance sustains hyperexcitability and promotes recurrent seizures.

Thus, the Gene Ontology (GO) enrichment analysis of genes expression was significantly modulated by the treatment, providing crucial insights into the molecular mechanisms underlying its observed anti-epileptic activity. This analysis showed a highly coordinated impact on key biological pathways, strongly implicating the regulation of kinase-driven signaling cascades and cellular stress responses.^27^ Molecular function (panel A) defines a reflective and statistically substantial enrichment of terms directly related to protein kinase activity and binding. The most critical enriched terms are the Protein serine/threonine kinase activity, MAP kinase kinase kinase activity, kinase binding, and protein kinase binding. This co-ordinated outline effectively recommends that the studied anticonvulsant candidates exhibit the potential to serve as broad modulator of kinase signaling networking. Considering the deep-rooted role of hyperactive kinase signaling, more specifically *via* the pathways such as MAPK, in promoting neuronal hyperexcitability and epileptogenesis,^28^ this outcome is of high importance. The suppressive findings of the mentioned kinase-related roles might be the possible mechanism necessary for the treatment plan to reduce anomalous neuronal signaling and unveil its anti-epileptic outcome. Cellular component (panel B) is the evident sign of enrichment in specific multi-protein complexes, as indicated by the terms, including insulin receptor complex and Alphav-beta3 integrin–IGF-1–IGF1R complex, which identifies their role in growth factor and integrin signaling pathways at the plasma membrane level. These kinds of complexes are well established to crosstalk with intracellular pathways which regulates cell survival, proliferation, and synaptic plasticity. In addition, enrichment of multivesicular body, internal vesicle can reveal the impact on endosomal sorting and mechanisms of protein degradation. The processes are essential for surface expression maintenance of receptors (such as neurotransmitter receptors) involved in regulation of neuronal excitability. Biological process (panel C) is the pictorial reflection of the results compiled in the other panels, pointing to the main functional importance. The most critical processes are peptidyl-serine phosphorylation and its positive regulation, which are fully in line with the kinase-centered MO function illustrated in panel A. The enrichment of the MAPK cascade and the transmembrane receptor protein tyrosine kinase signaling pathway further supports targeting of these particular, pro-epileptogenic signaling pathways.^29^ Moreover, the regulation of larger processes including response to stress regulation, cell population proliferation regulation, and regulation of intracellular signal transduction identifies that the candidates are able to cause pleiotropic events aside from immediate neuronal inhibition. These actions can possibly include the neuroprotection through reduction of cellular stress and regulation of glial cell proliferation, both of which are involved in epilepsy pathophysiology.

In conclusion, the GO enrichment analysis gives an integrative mechanistic account. The treatment's anti-epileptic activity is most probably mediated through a multi-faceted repression of major kinase signaling pathways (*e.g.*, MAPK), triggered at the level of membrane complexes, leading to changed phosphorylation events and ending in a diminished cellular stress response and normalized signal transduction. These data strongly place these candidates as a modulator of essential epileptogenic processes.

## 3. Conclusion

The studied series of 2,4,5 trichlorobenzenesulfonate based dihydrothiazoles 3(a–j) possess notable anticonvulsant activity, especially 3c. Potent, broad-spectrum anticonvulsant activity in both the 6 Hz psychomotor and PTZ-induced seizure models is strictly dependent on the presence of a cyclohexyl substituent at the thiazole N-3 position combined with an unsubstituted phenyl ring at C-4. Compound 3c, uniquely embodying this precise substitution pattern, exhibited complete protection in the 6 Hz model at 100 and 150 mg kg^−1^ (equivalent to levetiracetam) and full abolition of hind-limb extension and mortality in the PTZ model at 150 mg kg^−1^, with highly significant prolongation of all seizure phases even at 100 mg kg^−1^. This was supported by molecular docking and DFT studies that showed the strong binding of 3c to critical neurological targets, which agrees with its favorable *in vivo* results. Furthermore, network pharmacology provided a more comprehensive understanding of the mechanisms of the anticonvulsant activity. 3c and the entire series of 2,4,5 trichlorobenzenesulfonate based dihydrothiazoles are hence strong candidates for the development of new antiepileptic drugs. The strong agreement between *in vivo* efficacy and computational analyses supports the validity of the applied *in silico* approach. Although detailed experimental evaluation of solubility, blood–brain barrier permeability, and molecular target interactions was beyond the scope of this study, the observed central anticonvulsant activity provides functional evidence of adequate pharmacokinetic behavior. These findings highlight this scaffold as a promising starting point for further pharmacokinetic characterization and antiepileptic drug development.

## 4. Materials and methods

### 4.1. General information

All chemical reagents and solvents utilized in this study were sourced from various suppliers, including Sigma-Aldrich, Fluka, and Oakwood. Only analytical grade and solvents were employed throughout the investigation. Melting points were determined using the open-capillary method on a Fisher Johns electrical melting point apparatus. Thin-layer chromatography (TLC) was performed on precoated plates to monitor reaction progress and assess compound purity, with spot visualization under UV light. ^1^H NMR spectra were recorded on Bruker Ascend spectrometers operating at 400 MHz, while ^13^C NMR spectra were acquired at 101 MHz using DMSO-*d*_6_ as solvents. Tetramethyl silane (TMS) was used as the internal standard, and chemical shifts are reported in parts per million (ppm). Coupling constants (*J*) are given in hertz (Hz), and signal multiplicities are indicated as s (singlet), d (doublet), triplet (t), or m (multiplet).

### 4.2. Synthesis of 2,4,5-trichlorobenzenesulfonate based dihydrothiazoles

A series of novel 2,4,5-trichlorobenzenesulfonate based dihydrothiazoles 3(a–j) were synthesized *via* the condensation of 2,4,5-trichlorobenzenesulfonate thiosemicarbazone (1) with various phenacyl bromide (2). In this procedure, equimolar amount of the two reactants were dissolved in ethanol and refluxed for 24 hours, facilitating the formation of the desired dihydrothiazole core structure. The progress of the reaction was monitored by TLC until completion. Upon cooling, the resulting solid product was filtered off and washed with diethyl ether to remove impurities. The crude product was then recrystallized from ethanol to afford the pure 2,4,5-trichlorobenzenesulfonate based dihydrothiazoles derivatives in good yield.

#### 4.2.1 4-((*Z*)-(((*E*)-3,4-Diphenylthiazol-2(3*H*)-ylidene)hydrazineylidene)methyl)phenyl 2,4,5-trichlorobenzenesulfonate (3a)

Yield 82%, yellow color, mp 196–198 °C ^1^H NMR (400 MHz, DMSO-*d*_6_) *δ* 8.35 (1H, s), 8.15 (1H, s), 8.09 (1H, s), 7.76–7.68 (2H, m), 7.36 (2H, dd, *J* = 8.2, 6.5 Hz), 7.30–7.27 (2H, m), 7.26–7.21 (5H, m), 7.20–7.15 (3H, m), 6.67 (1H, s); ^13^C NMR (101 MHz, DMSO) *δ* 171.24, 150.24, 149.49, 140.21, 139.67, 137.90, 135.35, 134.65, 133.14, 132.88, 131.79, 131.75, 131.06, 129.39, 129.22, 129.10, 128.94, 128.80, 128.69, 128.46, 122.67, 102.29. Elemental anal. calcd for: C_28_H_18_Cl_3_N_3_O_3_S_2_: C, 54.69; H, 2.95; N, 6.83% observed: C, 54.99; H, 3.07; N, 6.93%.

#### 4.2.2 4-((*Z*)-(((*E*)-4-(4-Chlorophenyl)-3-phenylthiazol-2(3*H*)-ylidene)hydrazineylidene)methyl)phenyl 2,4,5-trichlorobenzenesulfonate (3b)

Yield 80%, yellow color, mp 233–235 °C ^1^H NMR (400 MHz, DMSO-*d*_6_) *δ* 8.35 (1H, s), 8.15 (1H, s), 8.09 (1H, s), 7.76–7.67 (2H, m), 7.38 (2H, dd, *J* = 8.2, 6.5 Hz), 7.34–7.26 (5H, m), 7.23–7.17 (4H, m), 6.73 (1H, s); ^13^C NMR (101 MHz, DMSO) *δ* 171.06, 150.42, 149.52, 139.67, 138.95, 137.70, 135.29, 134.65, 133.68, 133.14, 132.88, 131.79, 131.75, 130.57, 129.93, 129.49, 129.20, 129.13, 128.77, 128.60, 122.68, 103.04. Elemental anal. calcd for: C_28_H_17_Cl_4_N_3_O_3_S_2_: C, 51.79; H, 2.64; N, 6.47% observed: 51.89; H, 2.84; N, 6.54%.

#### 4.2.3 4-((*Z*)-(((*E*)-3-Cyclohexyl-4-phenylthiazol-2(3*H*)-ylidene)hydrazineylidene)methyl)phenyl 2,4,5-trichlorobenzenesulfonate (3c)

Yield 85%, yellow color, mp 175–177 °C ^1^H NMR (400 MHz, DMSO-*d*_6_) *δ* 8.29 (1H, s), 8.09 (1H, s), 7.80–7.73 (2H, m), 7.51 (3H, dd, *J* = 5.0, 1.9 Hz), 7.42 (2H, dd, *J* = 6.6, 3.0 Hz), 7.28–7.17 (2H, m), 6.32 (1H, s), 3.69 (1H, m), 2.60 (2H, d, *J* = 11.1 Hz), 1.69 (4H, dd, *J* = 28.4, 11.8 Hz), 1.51 (1H, d, *J* = 10.0 Hz), 1.11–0.94 (3H, m); ^13^C NMR (101 MHz, DMSO) *δ* 170.47, 149.28, 148.60, 141.42, 139.66, 135.69, 134.65, 133.15, 132.90, 132.15, 131.78, 129.76, 129.38, 129.32, 128.90, 122.65, 101.17, 59.18, 28.60, 26.05, 25.12. Elemental anal. calcd for: C_28_H_24_Cl_3_N_3_O_3_S_2_: C, 54.16; H, 3.90; N, 6.77% observed: 54.16; H, 3.90; N, 6.77%.

#### 4.2.4 4-((*Z*)-(((*E*)-4-(4-Chlorophenyl)-3-cyclohexylthiazol-2(3*H*)-ylidene)hydrazineylidene)methyl)phenyl 2,4,5-trichlorobenzenesulfonate (3d)

Yield 82%, light yellow color, mp 198–200 °C ^1^H NMR (400 MHz, DMSO-*d*_6_) *δ* 8.35 (1H, s), 8.30 (1H, d, *J* = 1.9 Hz), 8.09 (1H, s), 7.75 (2H, dd, *J* = 8.7, 2.7 Hz), 7.58 (2H, t, *J* = 8.4 Hz), 7.51–7.42 (2H, m), 7.22 (2H, dd, *J* = 8.7, 3.0 Hz), 6.36 (1H, s), 3.65 (1H, m), 2.59 (1H, d, *J* = 12.4 Hz), 1.70 (4H, dd, *J* = 22.3, 10.6)Hz, 1.60–1.40 (2H, m), 1.32–0.75 (4H, m); ^13^C NMR (101 MHz, DMSO) *δ* 170.38, 167.95, 149.62, 149.41, 149.31, 148.79, 142.88, 140.16, 139.65, 135.63, 134.64, 134.50, 133.29, 133.14, 132.90, 131.78, 131.21, 130.94, 129.40, 129.00, 128.93, 128.69, 128.18, 122.64, 101.92, 96.25, 59.30, 28.62, 25.98, 25.12. Elemental anal. calcd for: C_28_H_23_Cl_4_N_3_O_3_S_2_: C, 51.31; H, 3.54; N, 6.41% observed: C, 51.51; H, 3.64; N, 6.71%.

#### 4.2.5 4-((*Z*)-(((*E*)-3-Cyclohexyl-4-(4-hydroxyphenyl)thiazol-2(3*H*)-ylidene)hydrazineylidene)methyl)phenyl 2,4,5-trichlorobenzenesulfonate (3e)

Yield 85%, yellow color, mp 181–183 °C ^1^H NMR (400 MHz, DMSO-*d*_6_) *δ* 9.84 (1H, s), 8.35 (1H, s), 8.27 (1H, s), 8.09 (1H, s), 7.81–7.68 (2H, m), 7.27–7.12 (4H, m), 6.93–6.79 (2H, m), 6.17 (1H, s), 3.70 (1H, m), 2.61 (1H, s), 1.73 (2H, d, *J* = 10.8 Hz), 1.69–1.47 (3H, m), 1.06 (3H, dd, *J* = 11.6, 4.6 Hz); ^13^C NMR (101 MHz, DMSO) *δ* 170.47, 158.70, 149.21, 148.22, 141.66, 139.65, 135.78, 134.63, 133.14, 132.90, 131.77, 130.85, 128.82, 122.61, 116.00, 99.98, 58.89, 28.58, 26.07, 25.16. Elemental anal. calcd for: C_28_H_24_Cl_3_N_3_O_4_S_2_: C, 52.80; H, 3.80; N, 6.60% observed: C, 52.90; H, 3.89; N, 6.74%.

#### 4.2.6 4-((*Z*)-(((*E*)-3-Cyclohexyl-4-(*p*-tolyl)thiazol-2(3*H*)-ylidene)hydrazineylidene)methyl)phenyl 2,4,5-trichlorobenzenesulfonate (3f)

Yield 81%, yellow color, mp 206–208 °C ^1^H NMR (400 MHz, DMSO-*d*_6_) *δ* 8.35 (1H, s), 8.28 (1H, d, *J* = 1.9 Hz), 8.09 (1H, d, *J* = 1.4 Hz), 7.75 (2H, dd, *J* = 8.8, 2.7 Hz), 7.46 (1H, d, *J* = 8.0 Hz), 7.30 (3H, s), 7.25–7.17 (3H, m), 6.59 (1H, d, *J* = 273.6 Hz), 3.76–3.62 (1H, m), 2.61 (1H, d, *J* = 11.2 Hz), 2.51 (4H, p, *J* = 1.9 Hz), 1.79–1.61 (3H, m), 1.51 (2H, m), 1.14–0.90 (3H, m); ^13^C NMR (101 MHz, DMSO) *δ* 170.49, 168.17, 149.35, 149.26, 148.47, 141.47, 139.65, 139.31, 137.83, 135.72, 134.64, 133.14, 132.90, 131.78, 129.86, 129.25, 129.22, 128.94, 128.87, 126.11, 122.63, 100.77, 96.78, 59.07, 28.57, 26.04, 21.35, 21.14. Elemental anal. calcd for: C_29_H_26_Cl_3_N_3_O_3_S_2_: C, 54.85; H, 4.13; N, 6.62% observed: C, 54.96; H, 4.33; N, 6.81%.

#### 4.2.7 4-((*Z*)-(((*E*)-3-(2,6-Dimethylphenyl)-4-phenylthiazol-2(3*H*)-ylidene)hydrazineylidene)methyl)phenyl 2,4,5-trichlorobenzenesulfonate (3g)

Yield 75%, yellow color, mp 177–179 °C ^1^H NMR (400 MHz, DMSO-*d*_6_) *δ* 8.34 (1H, s), 8.13 (1H, s), 8.08 (1H, s), 7.71 (2H, d, *J* = 8.5 Hz), 7.22 (7H, m), 7.11 (4H, m), 6.76 (1H, s), 2.08 (6H, s); ^13^C NMR (101 MHz, DMSO) *δ* 169.54, 150.04, 149.43, 139.84, 139.66, 136.46, 136.28, 135.36, 134.62, 133.15, 132.83, 131.77, 130.64, 129.32, 129.25, 129.02, 128.92, 128.78, 127.87, 122.63, 102.14, 18.18. Elemental anal. calcd for: C_30_H_22_Cl_3_N_3_O_3_S_2_: C, 56.04; H, 3.45; N, 6.54% observed: C, 56.24; H, 3.62; N, 6.64%.

#### 4.2.8 4-((*Z*)-(((*E*)-3-(2,6-Dimethylphenyl)-4-(*p*-tolyl)thiazol-2(3*H*)-ylidene)hydrazineylidene)methyl)phenyl 2,4,5-trichlorobenzenesulfonate (3h)

Yield 78%, light yellow color, mp 169–171 °C ^1^H NMR (400 MHz, DMSO-*d*_6_) *δ* 8.34 (1H, s), 8.13 (1H, s), 8.08 (1H, s), 7.71 (2H, d, *J* = 8.5 Hz), 7.22 (7H, m), 7.11 (4H, m), 6.76 (1H, s), 2.08 (6H, s); ^13^C NMR (101 MHz, DMSO) *δ* 169.54, 150.04, 149.43, 139.84, 139.66, 136.46, 136.28, 135.36, 134.62, 133.15, 132.83, 131.77, 130.64, 129.32, 129.25, 129.02, 128.92, 128.78, 127.87, 122.63, 102.14, 18.18. Elemental anal. calcd for: C_31_H_24_Cl_3_N_3_O_3_S_2_: C, 56.67; H, 3.68; N, 6.40% observed: 56.87; H, 3.79; N, 6.50%.

#### 4.2.9 4-((*Z*)-(((*E*)-4-(4-Hydroxyphenyl)-3-(4-methoxyphenyl)thiazol-2(3*H*)-ylidene)hydrazineylidene)methyl)phenyl 2,4,5-trichlorobenzenesulfonate (3i)

Yield 76%, light yellow color, mp 250–252 °C ^1^H NMR (400 MHz, DMSO-*d*_6_) *δ* 9.63 (1H, s), 8.35 (1H, s), 8.10 (2H, d, *J* = 11.8 Hz), 7.70 (2H, d, *J* = 8.3 Hz), 7.17 (4H, dd, *J* = 18.7, 8.3 Hz), 6.94 (4H, dd, *J* = 31.6, 8.3 Hz), 6.61 (2H, d, *J* = 8.2 Hz), 6.44 (1H, s), 3.74 (3H, s); ^13^C NMR (101 MHz, DMSO) *δ* 171.65, 158.91, 158.02, 149.60, 149.37, 140.72, 139.66, 135.49, 134.64, 133.14, 132.88, 131.78, 131.75, 130.67, 130.40, 130.37, 128.98, 122.64, 121.84, 115.47, 114.50, 99.88, 55.75. Elemental anal. calcd for: C_29_H_20_Cl_3_N_3_O_5_S_2_: C, 52.70; H, 3.05; N, 6.36% observed: C, 52.82; H, 3.35; N, 6.66%.

#### 4.2.10 4-((*Z*)-(((*E*)-4-(4-Chlorophenyl)-3-isobutylthiazol-2(3*H*)-ylidene)hydrazineylidene)methyl)phenyl 2,4,5-trichlorobenzenesulfonate (3j)

Yield 75%, yellow color, mp 137–139 °C ^1^H NMR (400 MHz, DMSO-*d*_6_) *δ* 9.63 (1H, s), 8.35 (1H, s), 8.10 (2H, d, *J* = 11.8 Hz), 7.70 (2H, d, *J* = 8.3 Hz), 7.17 (4H, dd, *J* = 18.7, 8.3 Hz), 6.94 (4H, dd, *J* = 31.6, 8.3 Hz), 6.61 (2H, d, *J* = 8.2 Hz), 6.44 (1H, s), 3.74 (3H, s); ^13^C NMR (101 MHz, DMSO) *δ* 171.65, 158.91, 158.02, 149.60, 149.37, 140.72, 139.66, 135.49, 134.64, 133.14, 132.88, 131.78, 131.75, 130.67, 130.40, 130.37, 128.98, 122.64, 121.84, 115.47, 114.50, 99.88, 55.75. Elemental anal. calcd for: C_26_H_21_Cl_4_N_3_O_3_S_2_: C, 49.62; H, 3.36; N, 6.68% observed: C, 49.82; H, 3.46; N, 6.87%.

### 4.3. Synthetic compounds anticonvulsant potential

#### 4.3.1. Animals

Male C57BL/6 mice (18–25 g) bred and housed in 12 hour day and night cycle in animal facility at Faculty of Pharmacy, BZU Multan, were used in this study. Animals housed in strictly controlled standard conditions such as temperature (25° ± 2 °C) and humidity (40%), were prior acclimatized to experimenter's handling and testing environment to avoid their stress. All the animal experiments were approved by the Department of Pharmacology's Ethics Committee at BZU, Multan (17-PHL-S-24), and the “Institute of Laboratory Animal Resources” (ILAR) provided guidelines that were abided by Commission on Bioscience, National Research Council (NRC, 1996).

#### 4.3.2. Chemicals and drugs

Synthetic compounds (3a, 3b, 3c, 3d, 3e, 3f, 3g, 3h, 3i, 3j) were suspended in 1% Tween 80. While pentylenetetrazole (PTZ) 80 mg kg^−1^, diazepam (DZP) 5 mg kg^−1^ and levetiracetam (LEV) 50 mg kg^−1^ were dissolved in normal saline (Hafeez *et al.*, 2024). All chemical dilutions were prepared 10 ml kg^−1^ for administering intraperitoneal (i.p) injections. All liquid preparations were freshly prepared and administered according to the weight of mice by using calculation formula (Rehman *et al.*, 2024); volume to be administered (ml) = required dose (mg kg^−1^) *x* mice weight (kg)/available concentration (mg ml^−1^).

#### 4.3.3. 6 Hz seizure model

In 6 Hz seizure testing model, synthetic compounds (100 mg kg^−1^) were administered 40–45 minutes prior to 6 Hz corneal stimulation at 6 Hz frequency with pulse width of 0.2 ms and 32 mA action potential for 3 s. Animals were divided among 10 synthetic compounds and one standard treatment group. Before 6 Hz shock delivery, 1% lidocaine drop was applied in mouse corneas for better shock delivery. Stereotyped behavior with stun position, twitching of vibrissae, forelimb clonus, Straub's tail, grooming and rearing was observed and animal was considered as fully protected if it returned to its normal baseline behavior within 10 s after applying shock. Psychomotor seizure comparison was observed among synthetic compounds treated groups and standard group receiving LEV 50 mg kg^−1^ (Tahmasebi *et al.*, 2024).

#### 4.3.4. PTZ seizure model

Mice were divided into 11 groups. 10 groups received synthetic compounds 100 mg kg^−1^ and one group received normal saline. After 40–45 minutes of respective treatment chemoconvulsant PTZ 80 mg kg^−1^ was injected to all the groups. Latencies of myoclonic jerk, tonic–clonic seizure, hind limb extension and death were observed right after PTZ injection for 30 minutes. Animals were considered protected if they survived for 30 minutes after PTZ injection (Rasool *et al.*, 2023).

#### 4.3.5. Stereotaxic surgery and EEG

Before electrode placement mice were injected i.p ketamine (87.5 mg kg^−1^) xylazine (12.5 mg kg^−1^) cocktail. Mice unconsciousness was observed by toe punching withdrawal reflex. Animal head was fixed on stereotaxic frame (Stoelting, USA) with adjustable arms, was shaved to remove fur and cleansed with 70% alcohol. Stereotaxic stage was supplemented with thermoregulating heating pad to regulate temperature at 27–30 °C. Eye ointment was applied to avoid corneal desiccation and irritation. 2 cm mid-line head skin incised with surgical blade and retracted with bulldog clamps. To clean and dry exposed skull hydrogen peroxide was applied. Tripolar cortical electrode was implanted on already drilled holes while protecting meningeal layers of brain, at coordinates from bregma point AP + 2 mm and LL ± 1.5 mm. Acrylic dental cement slurry was used to intact the electrode firmly and 0.9% normal saline was injected subcutaneously to animal to avoid dehydration. Animal was given 5–7 days recovery period (Shakoor *et al.*, 2024).

**Fig. 11:**
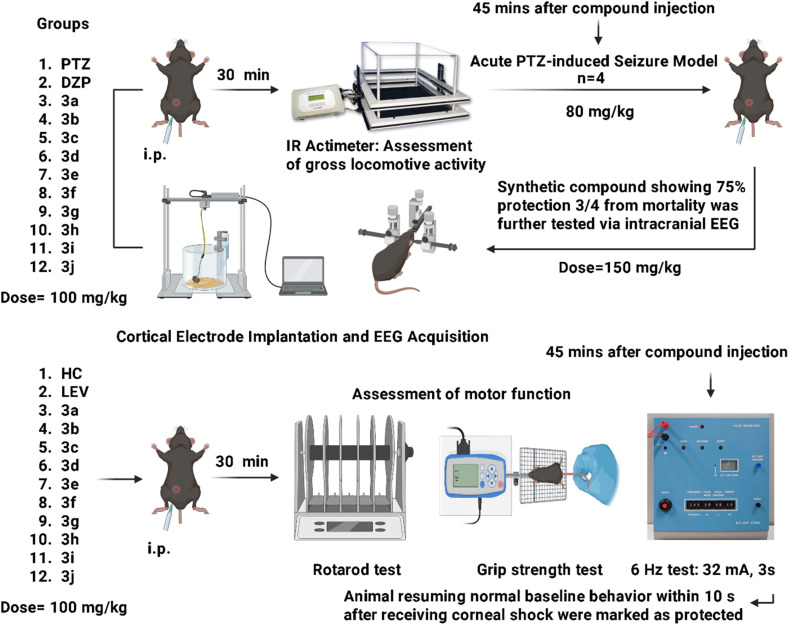
Experimental layout of synthetic compounds tested in acute PTZ-induced seizure and 6 Hz seizure models. IR actimeter, rotarod and grip strength tests were performed before seizure models. 3c compound showed 100% protection at 150 mg kg^−1^ and was assessed with EEG acquisition. Figure panel was created using https://BioRender.com (RO29EFQRLF, dated February 24th, 2026).

After healing span animal came to normal life and EEG was recorded by using analog–digital converter (PowerLab 8/30 ML870, ADInstruments) and 8-channel bioamplifiers (ADInstruments Ltd, Sydney, Australia) while video was recorded by using Logitech camera on laptop. Animal was acclimatized in plexiglass EEG cage and baseline was recorded for 20–30 minutes before giving synthetic compound 3c 150 mg kg^−1^ i.p. After 40–45 minutes PTZ 80 mg kg^−1^ was administered and EEG was recorded for 30 minutes with a signal sampling rate of 200 Hz and bandpass filtered between 0.1 and 60 Hz. Electroencephalogram spike changes were analyzed with Muneeb *et al.*'s published method (Anjum *et al.*, 2018). It was explained in experimental plan Fig. 11.

#### 4.3.6. Locomotor activity test

Animal was placed in open arena (45 × 45 cm) center of open field apparatus connected with Actimeter (Panlab Harvard apparatus IR Actimeter, Barcelona, Spain) having upper and lower 16 photobeam sensors. Slow rearing (SR) and fast rearing (FR) as vertical locomotor activity and slow movement (SM) and fast movement (FM) as horizontal locomotor activity were sensed and recorded by control unit when animal interrupted photobeam sensors (Malik *et al.*, 2023). This test was performed at maximum doses used in seizure models.

#### 4.3.7. Grip strength test

Forelimb grip strength was measured by using grip strength meter. Mouse was allowed to grasp the bar with forelimbs and gently pulled back by the tail. Grip strength was recorded by the connected transducer in grams as a peak pull force per grip. Test was performed 6 times consecutively with one minute gap and average was used for each and every animal. In test the readings of animals who griped with single fore paw or with hind limbs were excluded. This test was performed at maximum doses used in seizure models (Takeshita *et al.*, 2017).

#### 4.3.8. Motor co-ordination test

Mice were tested for motor co-ordination by using mouse rotarod (UGO Basile, Italy; cat. no. 47600) after respective treatment at maximum doses used in seizure models, according to the method described. Rotarod treadmill consists of long rod having 3 cm diameter guarded by two round plates separating the apparatus into 5 chambers allowing 5 animals to be tested in same time. Mice were placed on rod rotating at speed 4 rpm that was increased to 40 rpm for 5 minutes and latencies of mice falling from rod were noted (Noda *et al.*, 2020).

#### 4.3.9. Statistical analysis

Graph Pad Prism version 9.5 was used for statistical analysis and one way ANOVA *post hoc* Dunnett test was applied for multiple comparison. To show significance of the results data was represented with mean ± SD and *P* < 0.05. *P* values less than 0.05 indicated significant difference while *F* value indicated variability between the intragroup and intergroups. Descriptive statistics analysis indicated mean and standard deviation of each and every group (Bertinetto *et al.*, 2020).

### 4.4. Molecular docking

The three dimensional structure of the chosen target receptor, *i.e.* of human GABAA crystallographic structure with PDB ID 6X3W has been retrieved from RCSB Protein Data Bank.^30^ It was processed by employing AutoDockTools-1.5.7 where the co-crystallized ligand along with the water molecules were deleted followed by addition of Kollman charges followed by the addition of polar hydrogens.

While GABAA contains different binding sites (each of which is more or less favorable for a particular drug), one always needs to look in order to examine interactions with these different subunits. 3D structure of GABAA having PDB ID 6X3W contains two α1 (B–D chains), two β2 (A–C chains), along with one γ2 (E chain) subunits. Although classical benzodiazepines are familiar to bind at the interface of α–γ near the residues of a1H101, a1Y159, a1T206, and a1Y209,^31^ however recent literature^32^ reported its ability to get bound in three other sites at α1 (chain B)–β2 (chain A), α1 (chain D)–β2 (chain C) and β2 (chain C)–γ2 (chain E) interfaces. Barbiturates bind α1 (chain B)–β2 (chain A) and β2 (chain A)–γ2 (chain E). All these five (A, B, C, D, and E) possible binding sites were thus retained for further studies.

The 2D structures of series 3(a–j) were sketched out *via* ChemDraw 20.1.1 (ref. 24) and Chem3D 20.1.1 facilitated in energy minimization by incorporating the MM2 force field.^33^ The attained optimized conformation of this series of compounds were eventually saved in pdb file format. The pdbqt file format of these conformations of this series of compounds was fetched with the aid of MGL tools, as it is mandatory to perform docking experiment. Autodock vina^34^ assisted in molecular docking and the best docking pose of each ligand was visualized *via* the Biovia Discovery Studio visualizer.^35^ These studies are executed to establish a comparison as well as visualize the interactions of these compounds with the experimental activities with a view to better estimate their binding mechanisms.

**Fig. 12:**
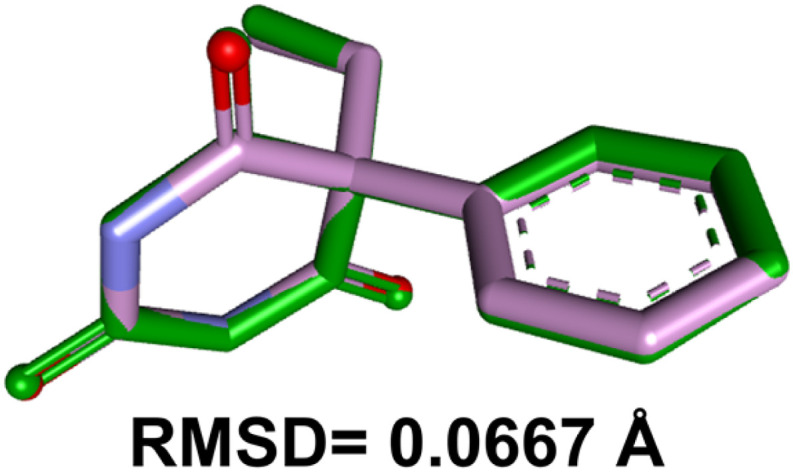
The superimposed poses of the pre-docked (green) and re-docked (pink with blue hue) co-crystallized ligand of the target protein (PDB ID 6X3W).

In order to exercise the validation of molecular docking method, we opted a re-docking experiment of the co-crystallized ligand of the target protein into its binding site. Upon execution, it was seen that the re-docked co-crystallized ligand had a root mean-square deviation (RMSD) value of 0.0667 Å against its respective native co-crystallized pose before docking. It has clearly indicated that the visualized binding interactions are contributed by their natively attached co-crystallized ligand. RMSD value (<2.0 Å)^36^ confirmed the accuracy and reliability of employed docking protocol to evaluate the binding interactions of the synthesized compounds 3(a–j). The superimposition of both the re-docked and the natively adhered co-crystallized poses is portrayed in Fig. 12.

### 4.5. Drug-likeness and BBB permeability prediction

The relevant data of *in silico* drug-likeness and BBB permeation of the series of studied compounds 3(a–j) was predicted with the courtesy of Molsoft webserver https://molsoft.com/mprop/.

### 4.6. DFT calculations

Density functional theory (DFT) approach was used to evaluate the bioactivity of compounds by examining their reactivity, molecular structures, and their associated interactions with biological targets. The 3-dimensional structures of the studied compounds were initially built using GaussView 6.0, followed by full geometry optimization with the Gaussian 16 software suite.^37^ All calculations were conducted in the gas phase incorporating the B3LYP functional, and employing 6-311G (d,p) basis set for N, C, O, and H atoms.^38,39^ To confirm that the geometries optimized were actual local minima on the potential energy surface, vibrational frequency analyses was conducted. Besides structural information, electronic properties were investigated by the calculation of frontier molecular orbitals (FMOs), in this case, the highest occupied molecular orbital (HOMO) and the lowest unoccupied molecular orbital (LUMO). These orbitals are considered critical to evaluate key quantum chemical descriptors such as chemical reactivity, electrophilicity, chemical softness, chemical hardness, electron affinity and ionization potential, which were inferred from the HOMO/LUMO energy gap (Δ*E*).^40,41^

Global reactivity descriptors, including electron affinity (*A*), ionization potential (*I*), chemical hardness (*η*), chemical softness (*σ*), electronic chemical potential (*µ*), and global electrophilicity index (*ω*)^42,43^ for these ligands, were calculated using the following eqn (1)–(5):

1 *A* = −*E*_LUMO

2 *I* = −*E*_HOMO

3 *η* = (*I* − *A*)/2

4 *σ* = 1/*η*

5 *µ* = −(*I* + *A*)/2

6 *ω* = *µ*^2^/(2*η*)

In addition, further investigational analysis of molecular premises which are fairly engaged in nucleophilic or electrophilic attacks, Molecular Electrostatic Potential (MEP) surfaces were also rendered.

### 4.7. Network pharmacology

The SMILES string of all ligands were fed to the Swiss Target Prediction web server^44^ to acquire the data of potential gene targets of the series 3(a–j). The genes accountable for epilepsy are downloaded from the GeneCards database,^45^ keeping a GIFt score cut-off of 67, to ensure their biological significance.

The string database aids effectively in the networking of obtained gene expressions with their relevant pathways.^46^ The visualization of these results was carried out employing Cytoscape v.3.10.3.^47^ Supplement to it, Gene Ontology (GO) enrichment analysis was also executed across the three domains (Biological Process (BP), Molecular Function (MF), and Cellular Component (CC)) with the aid of the ShinyGo 0.85 software,^48^ to efficiently and keenly interpret the transcriptional processes.

## Author contributions

Faiqa Noreen, Muhammad Iftikhar Shoukat, Farhan Siddique: investigation, formal analysis, software. Sumaira Nadeem, Xianliang Zhao: formal analysis, software, data curation, investigation. Mostafa A. Ismail, Magdi E. A. Zaki, Sobhi M. Gomha: formal analysis, resources, funding acquisition. Imran Imran, Zahid Shafiq: writing–original draft, supervision, conceptualization.

## Conflicts of interest

There are no conflicts to declare.

## Supplementary Material

RA-016-D6RA00305B-s001

## Data Availability

The data used for the manuscript entitled “Design, synthesis and anticonvulsant evaluation of novel 2,4,5-trichlorobenzenesulfonate-based dihydrothiazoles supported by *in vivo* and *in silico* studies” will be included in an supplementary information (SI), available online on RSC Advances web site. Supplementary information is available. See DOI: https://doi.org/10.1039/d6ra00305b.
