# Supplementary material for: Design, synthesis and anticonvulsant evaluation of novel 2,4,5-trichlorobenzenesulfonate-based dihydrothiazoles supported by in vivo and in silico studies
Source: RSC Adv. 2026 Mar 3;16(13):12100–18. doi: 10.1039/d6ra00305b (PMC12956243; doi:10.1039/d6ra00305b)

Supplementary Information (SI) for RSC Advances.
This journal is © The Royal Society of Chemistry 2026

^{1}H and ^{13}C NMR of 3a

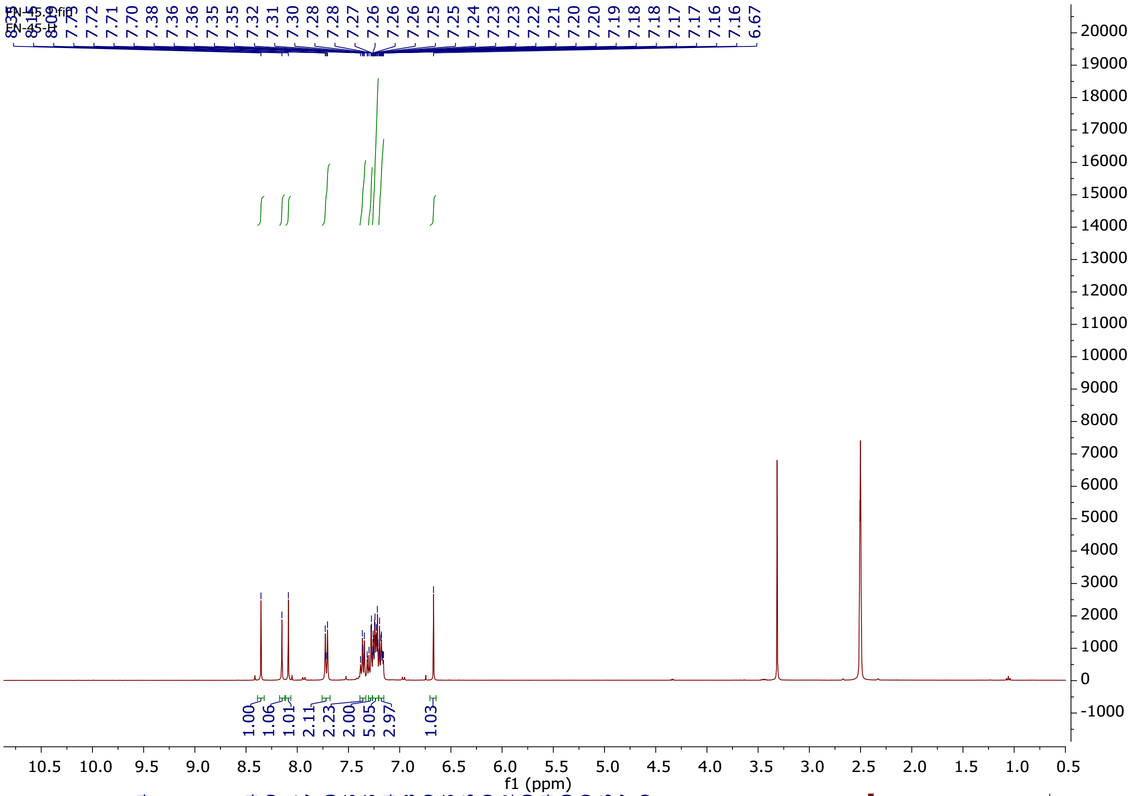

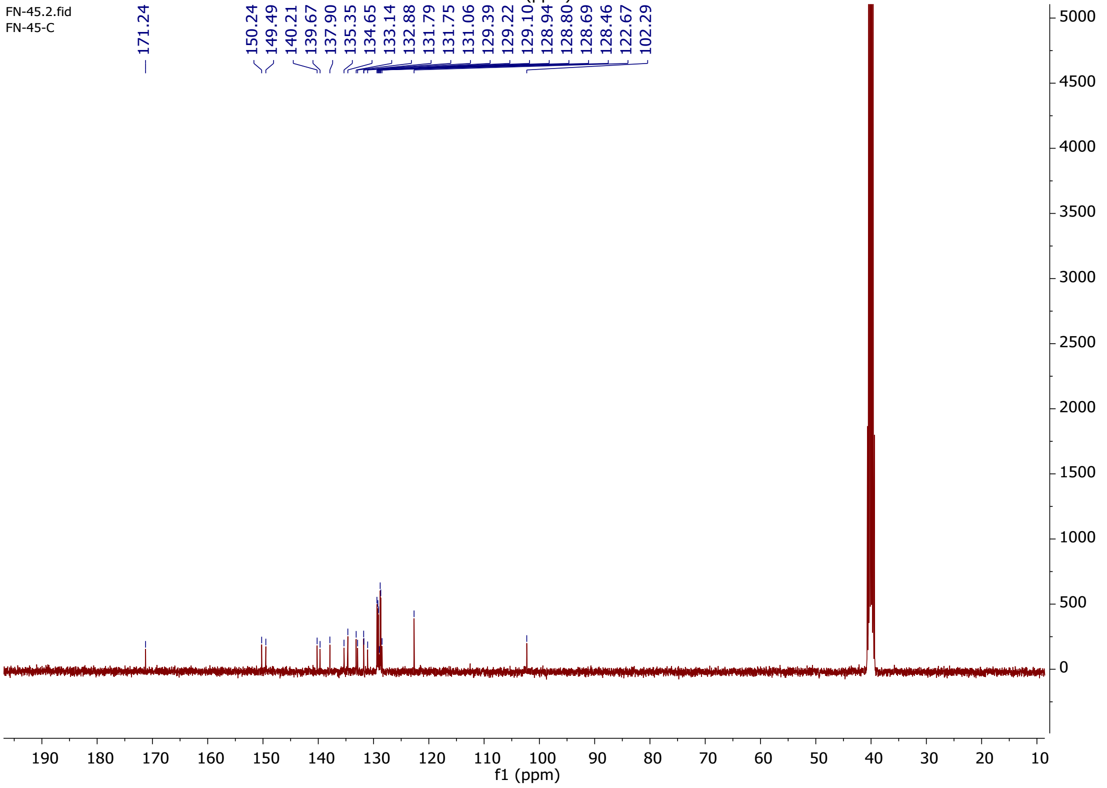

^{1}H and ^{13}C NMR of 3b

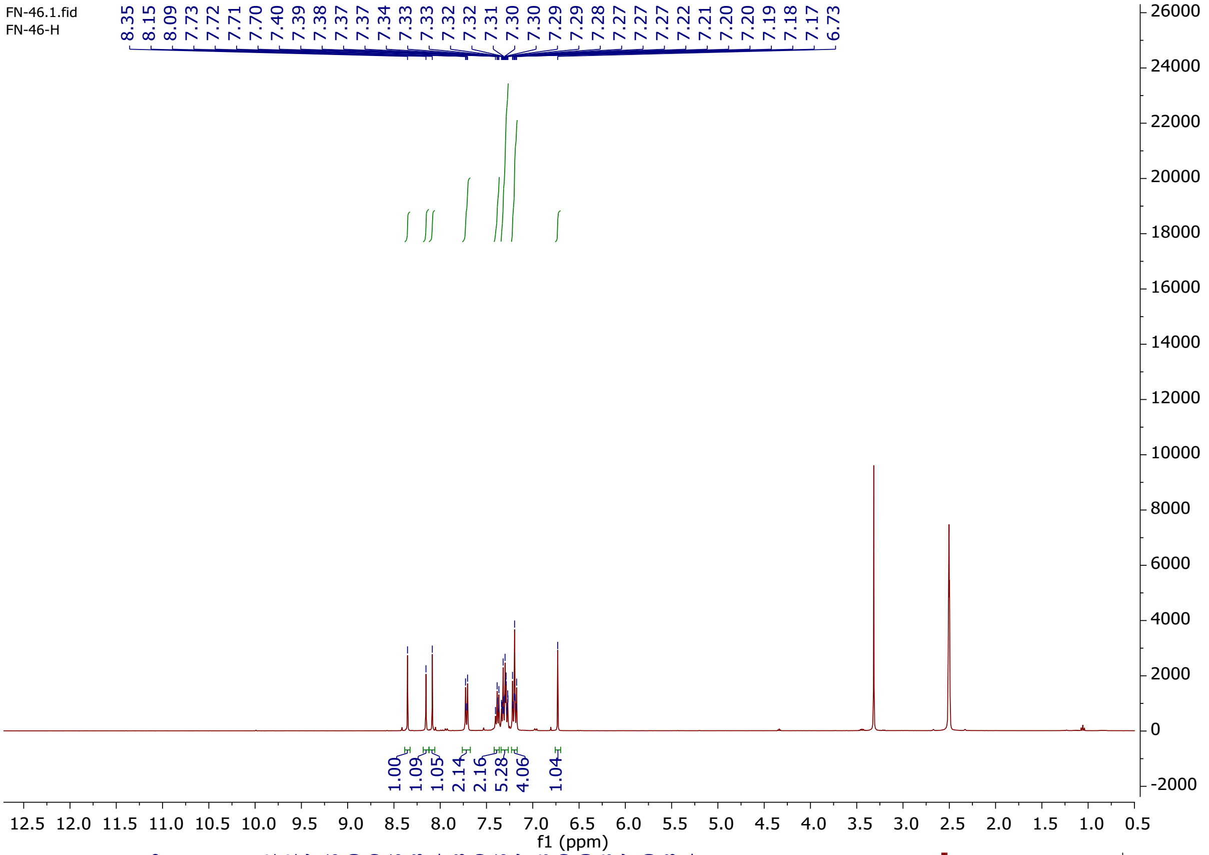

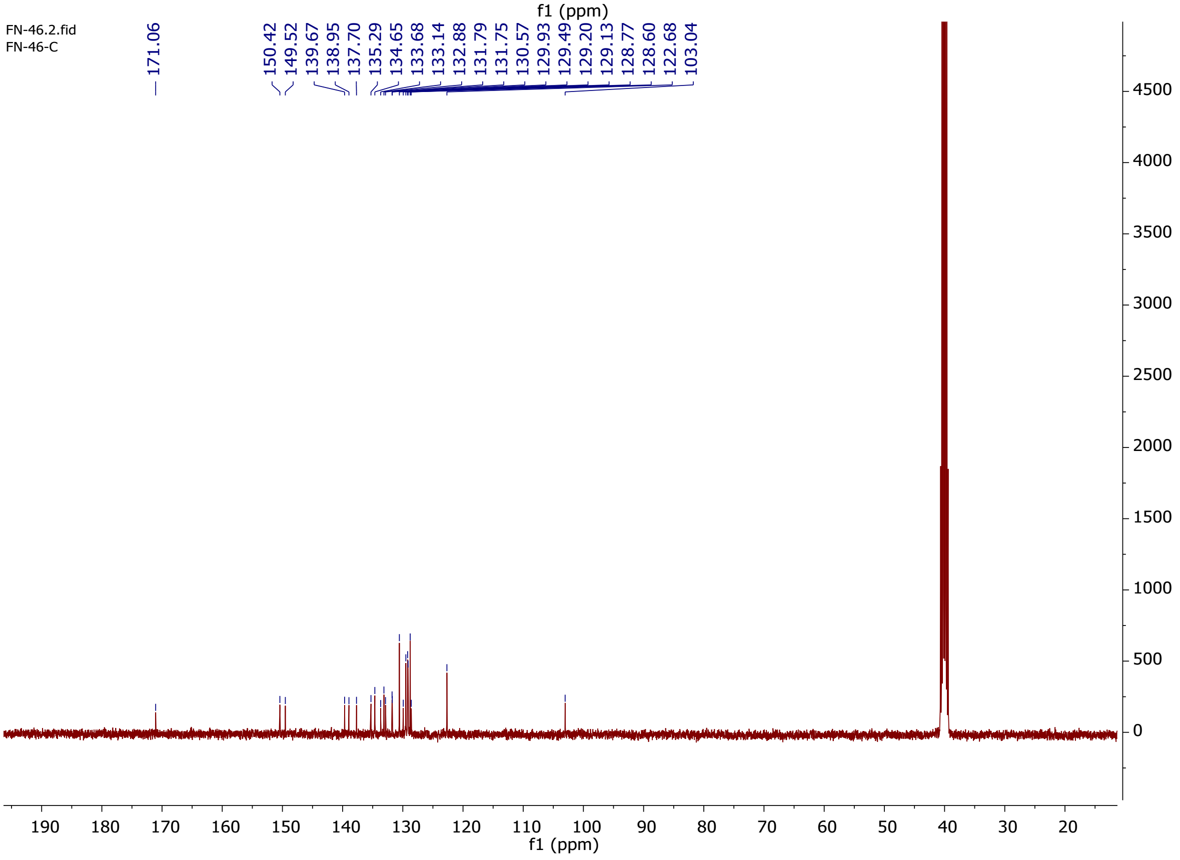

^{1}H and ^{13}C NMR of 3c

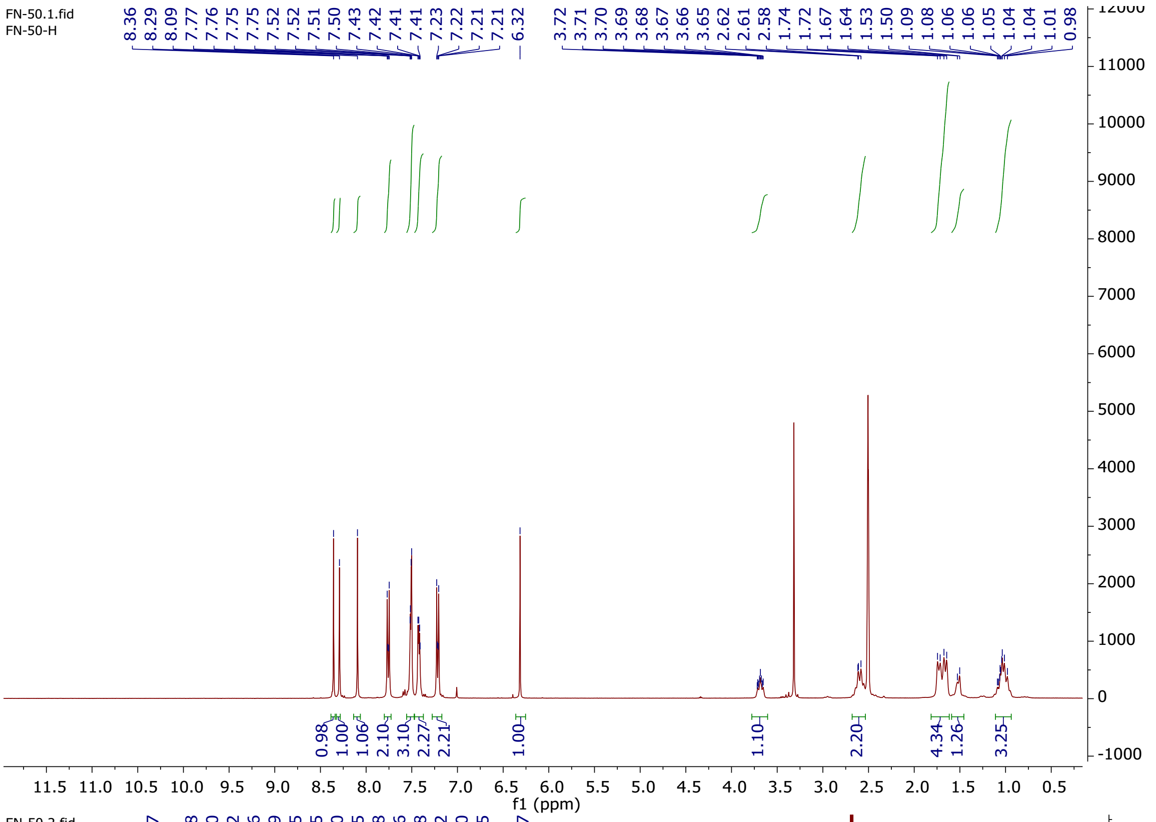

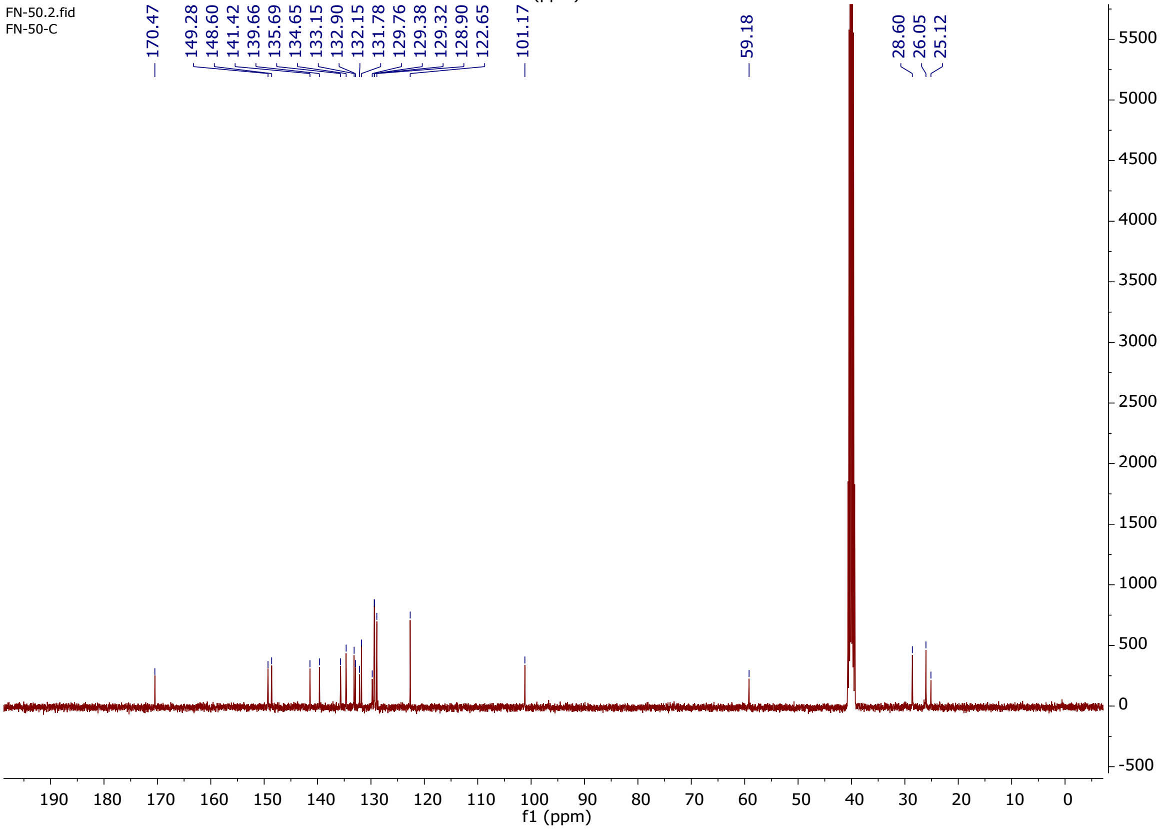

^{1}H and ^{13}C NMR of 3d

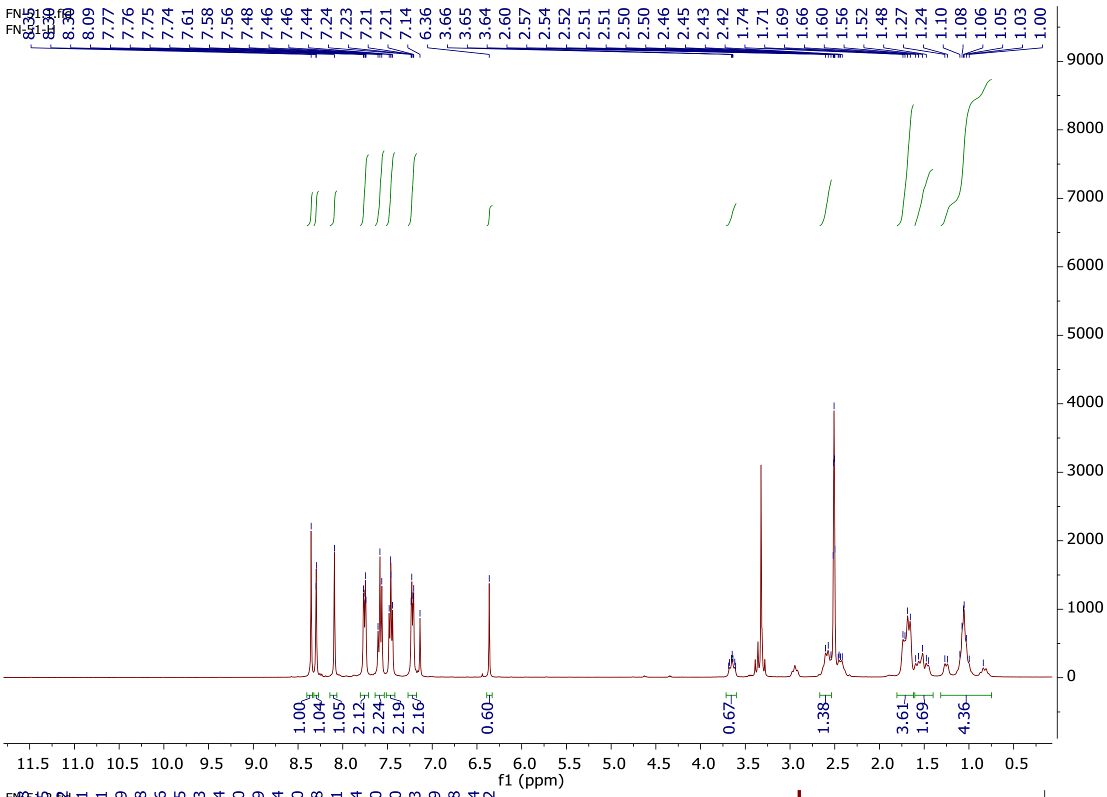

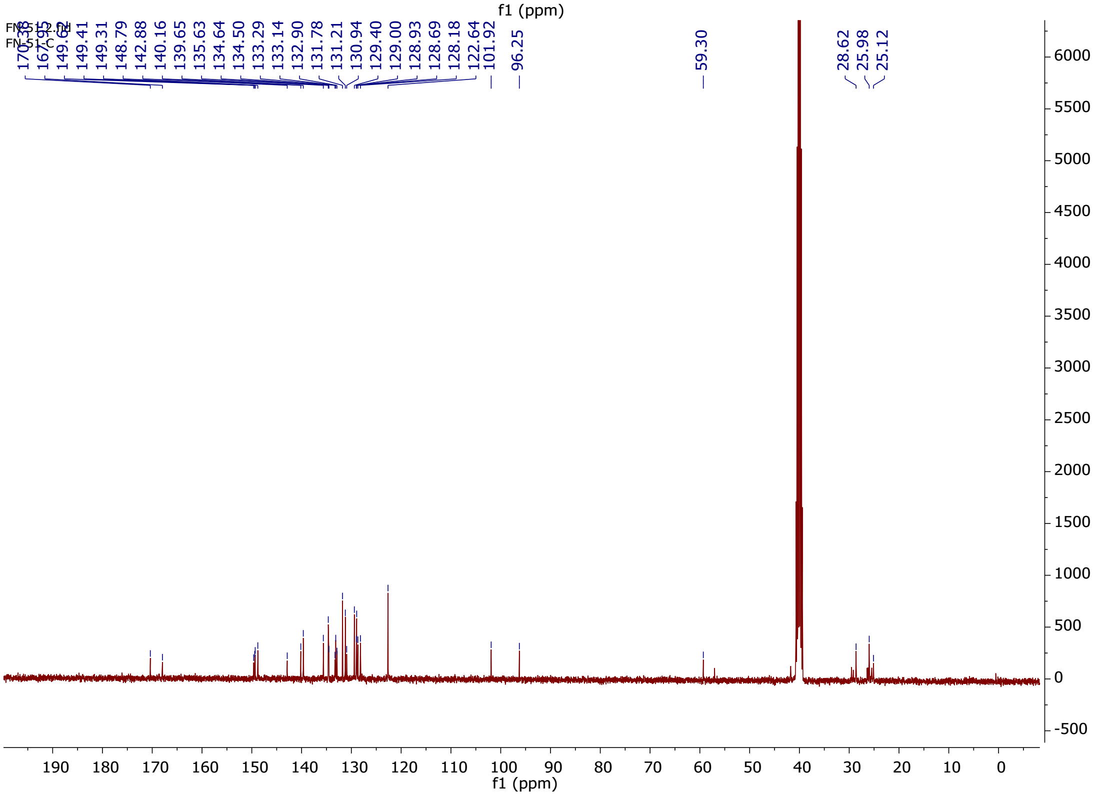

^{1}H and ^{13}C NMR of 3e

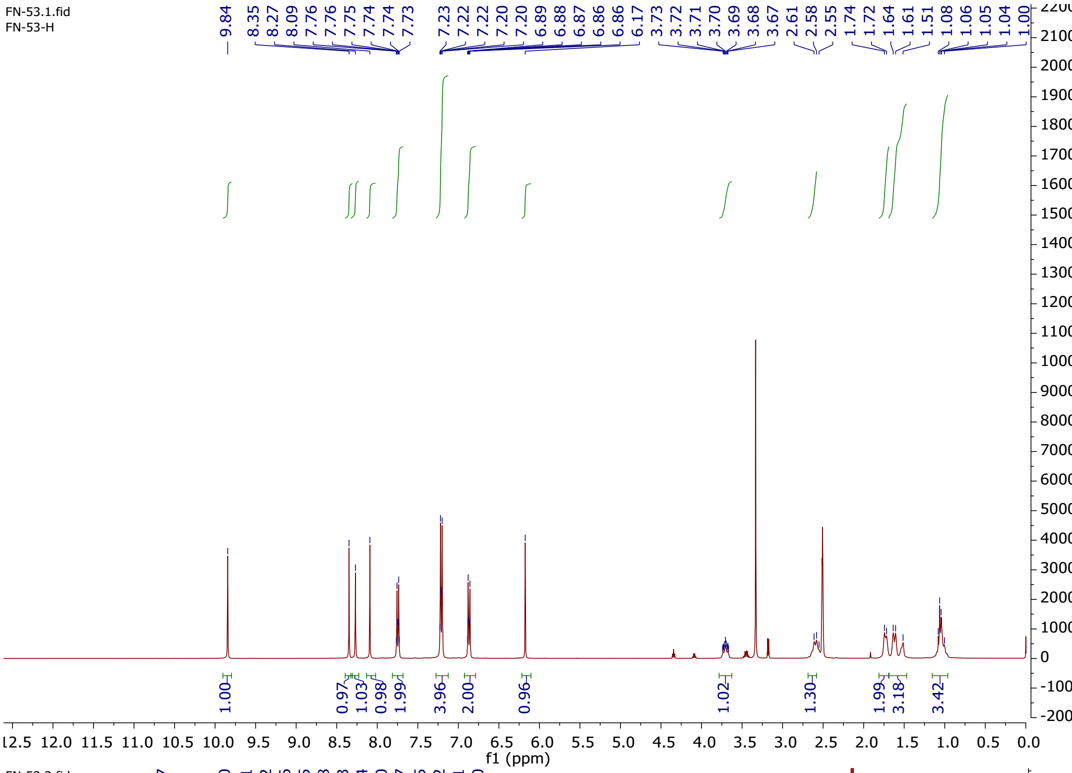

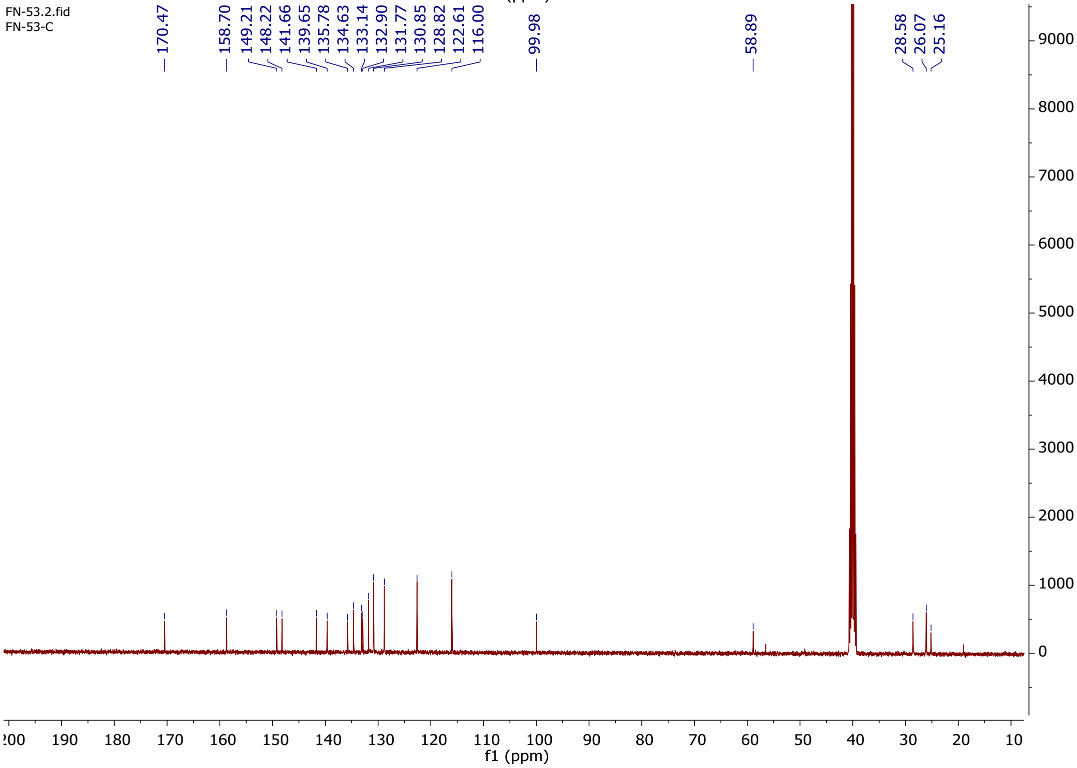

^{1}H and ^{13}C NMR of 3f

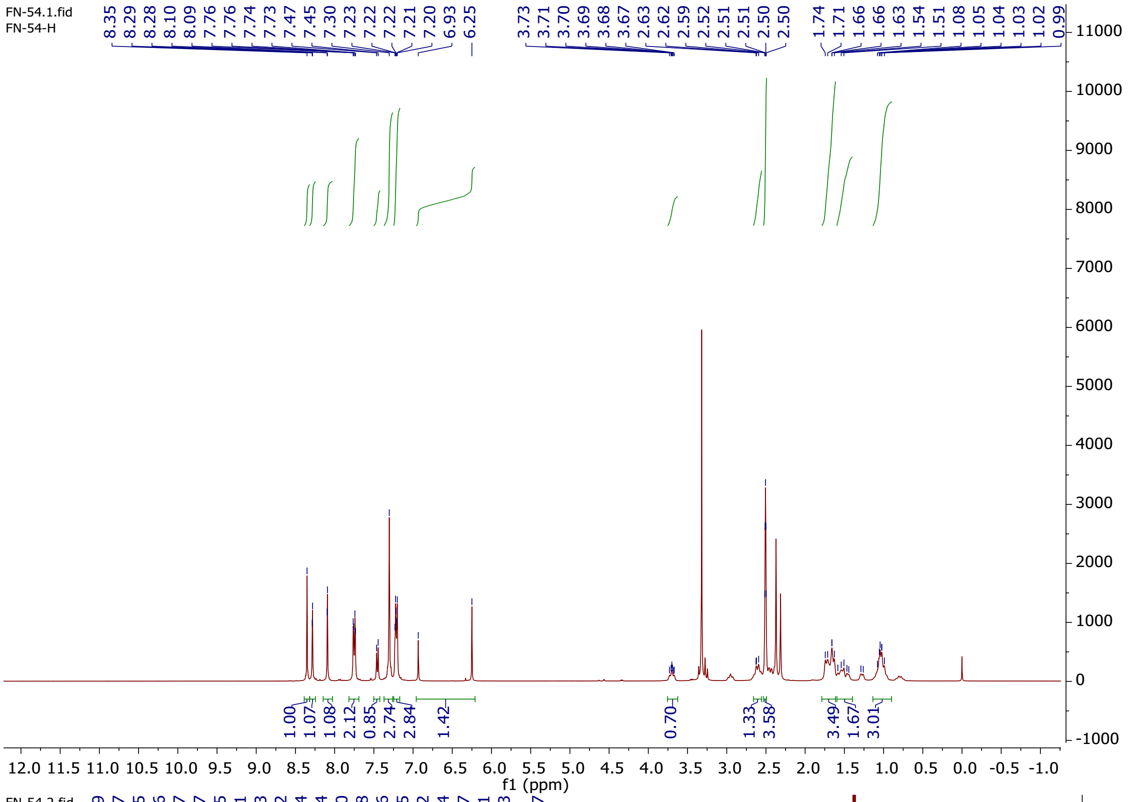

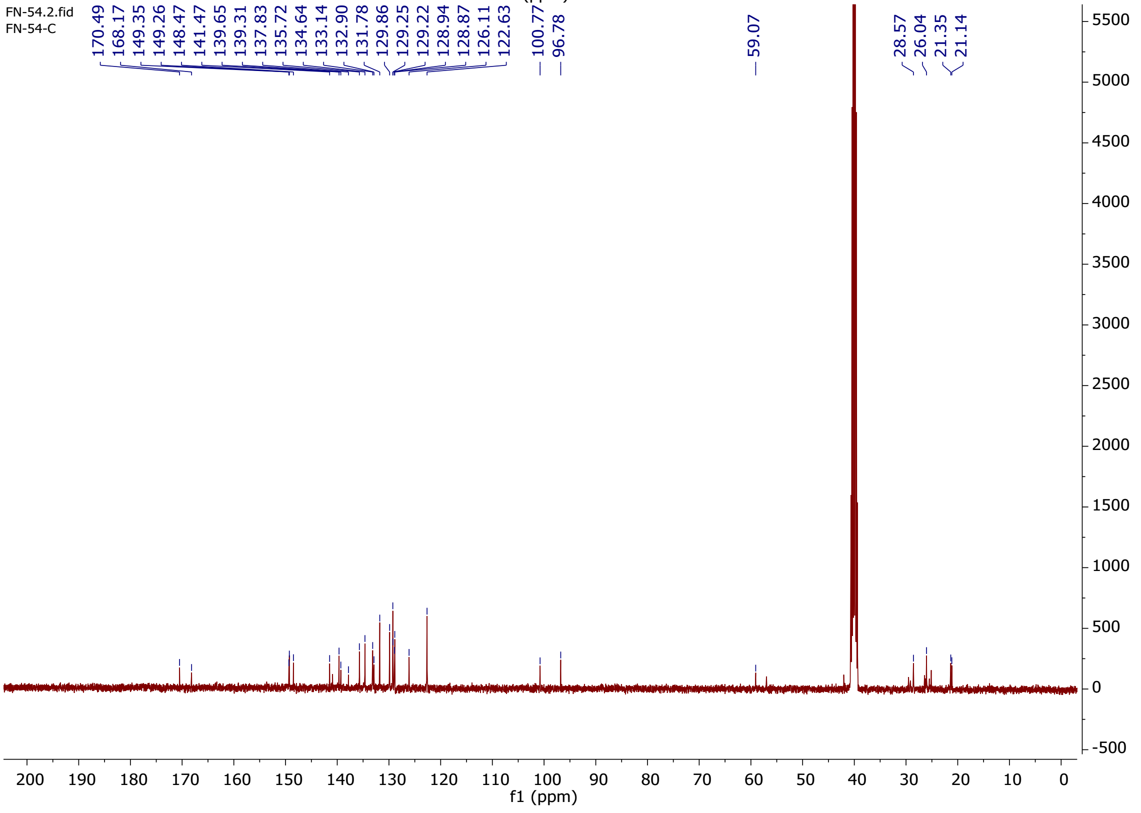

^{1}H and ^{13}C NMR of 3g

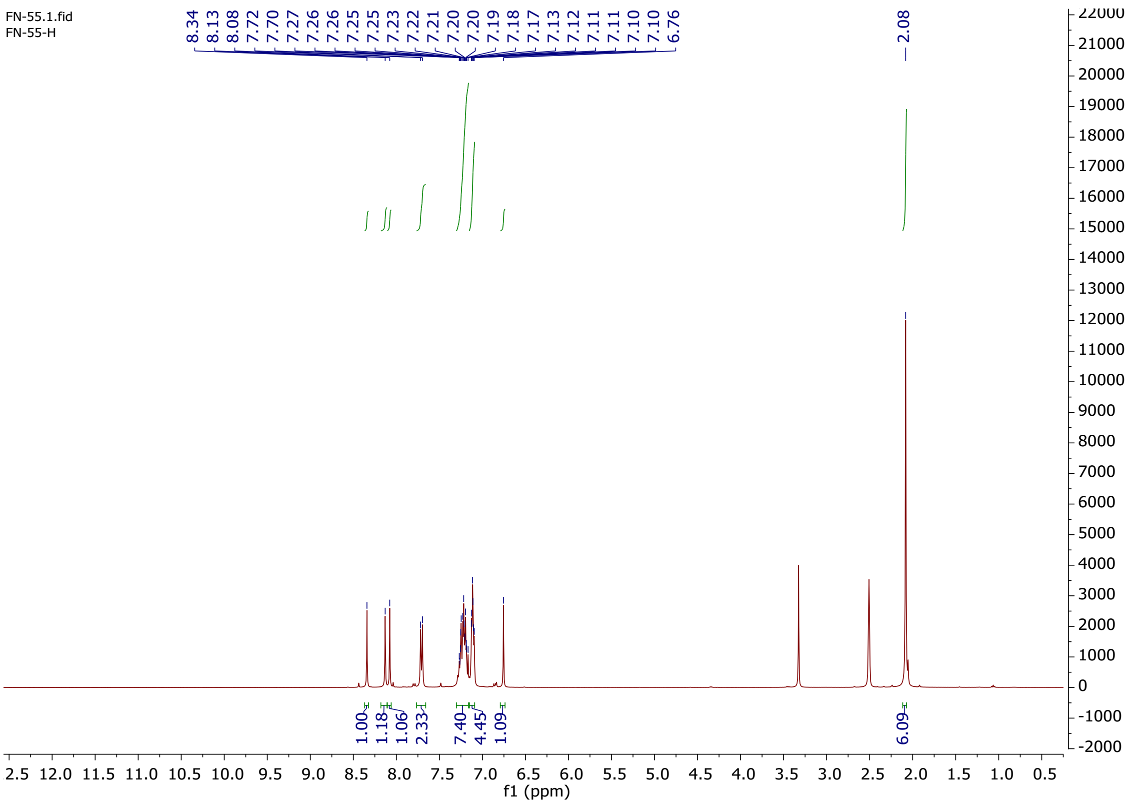

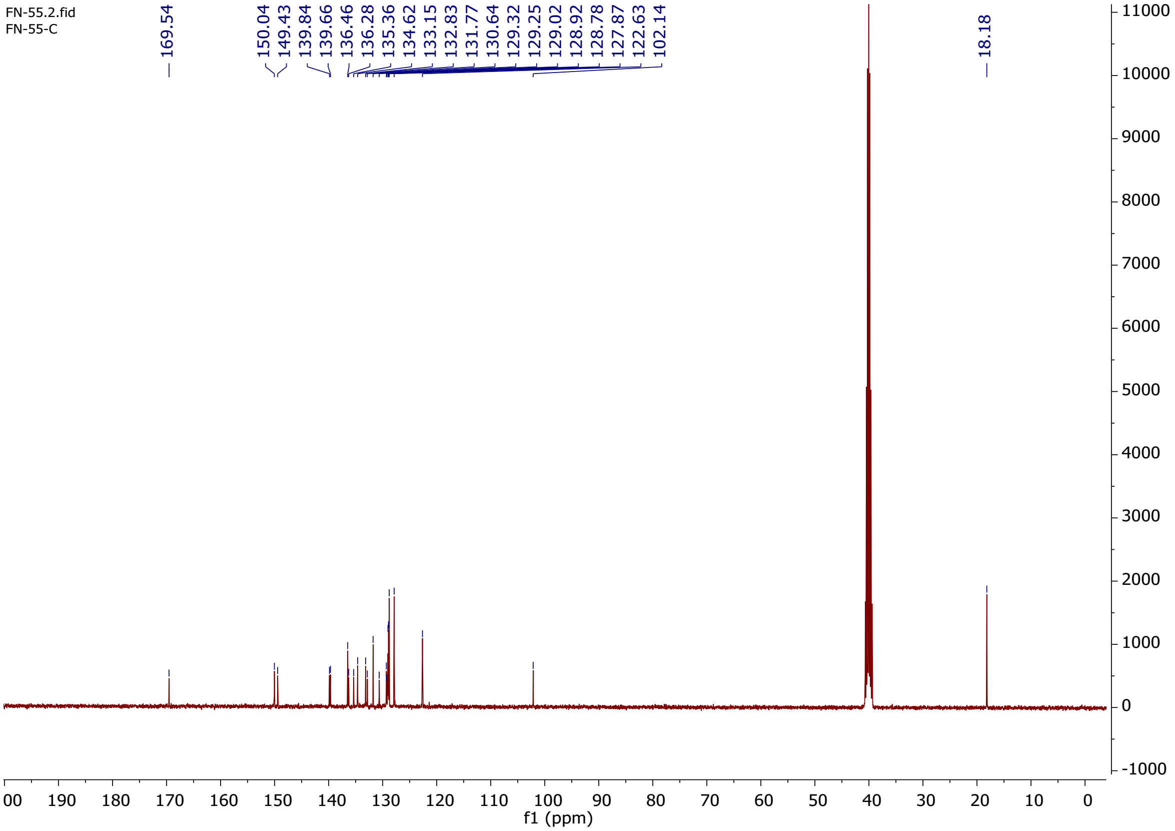

^{1}H and ^{13}C NMR of 3h

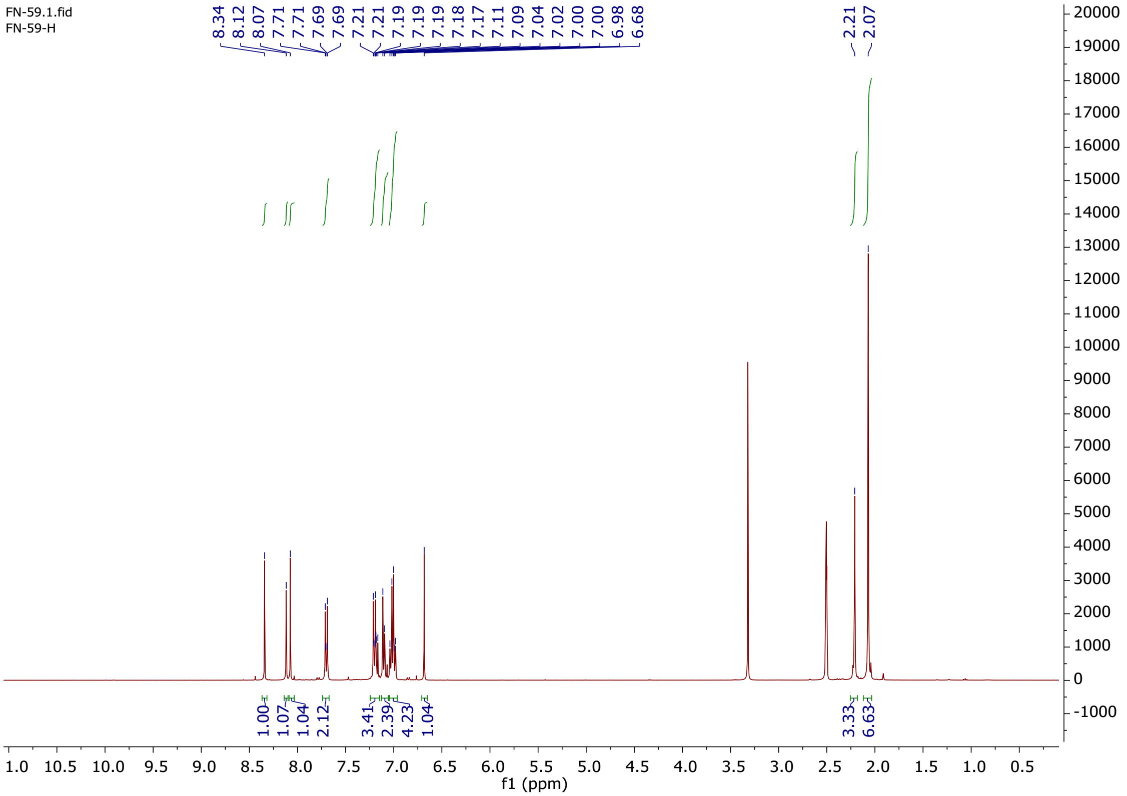

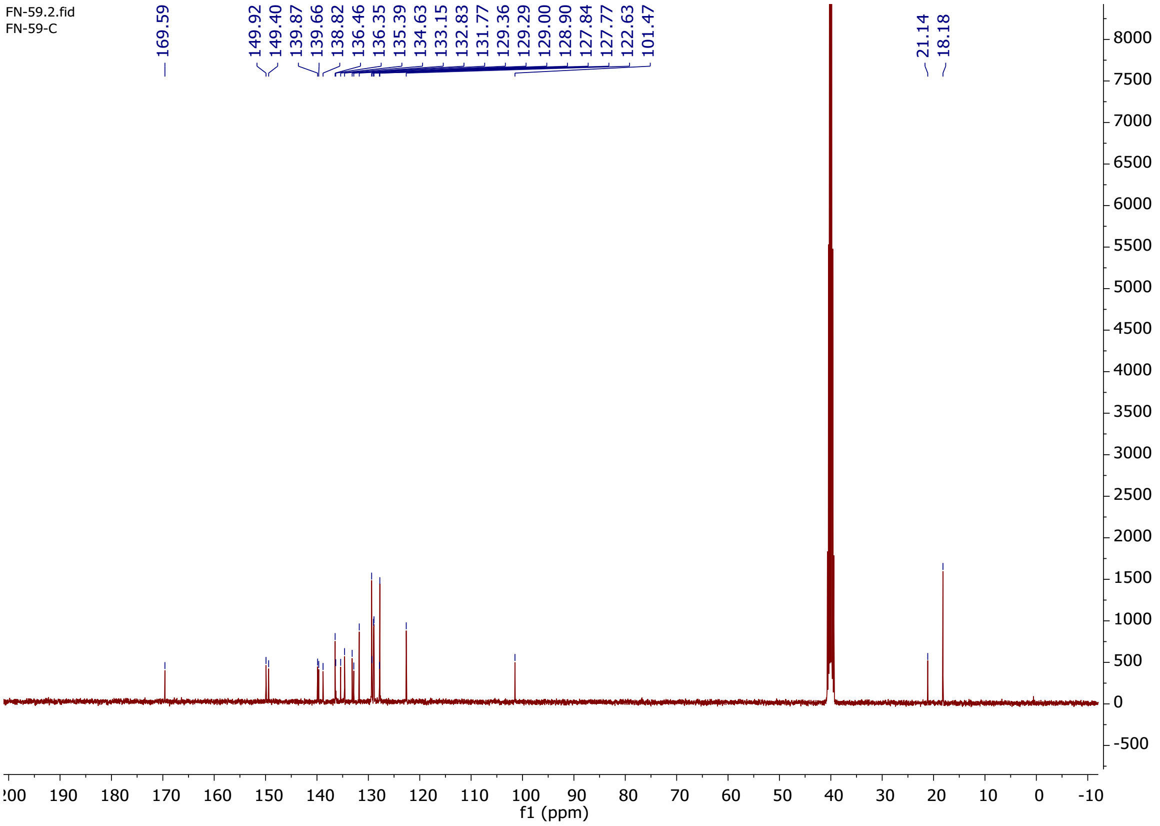

^{1}H and ^{13}C NMR of 3i

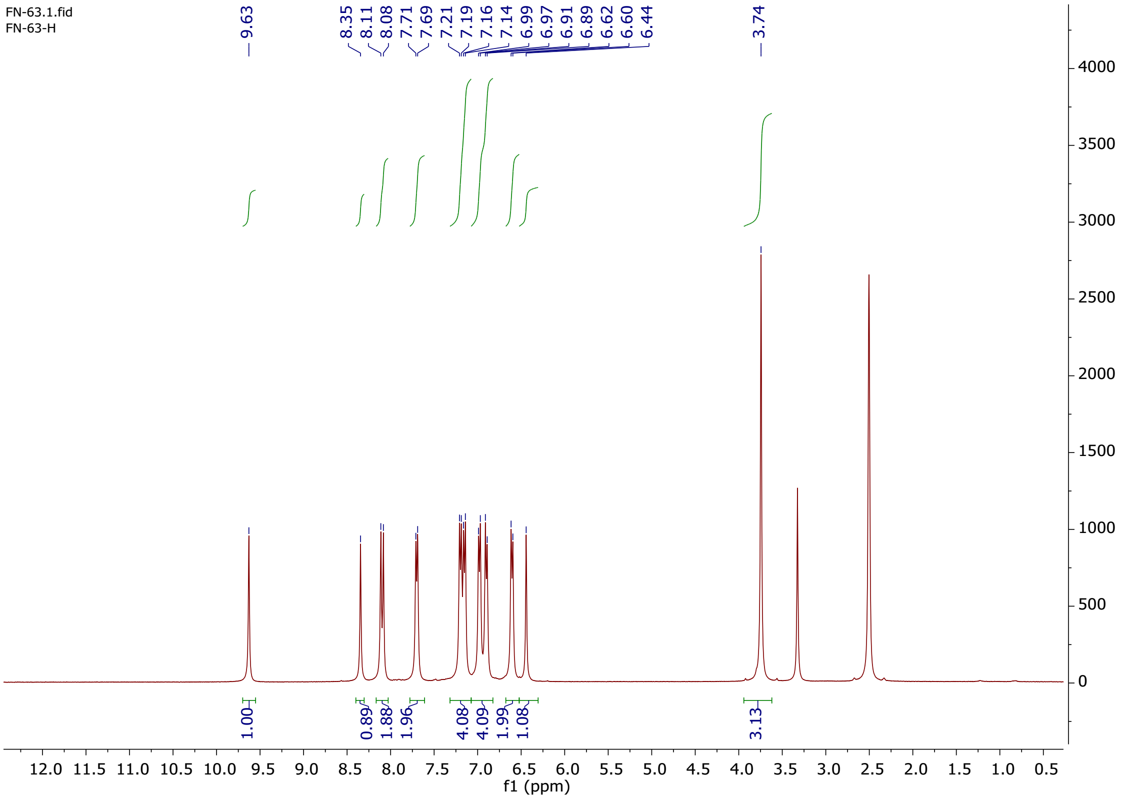

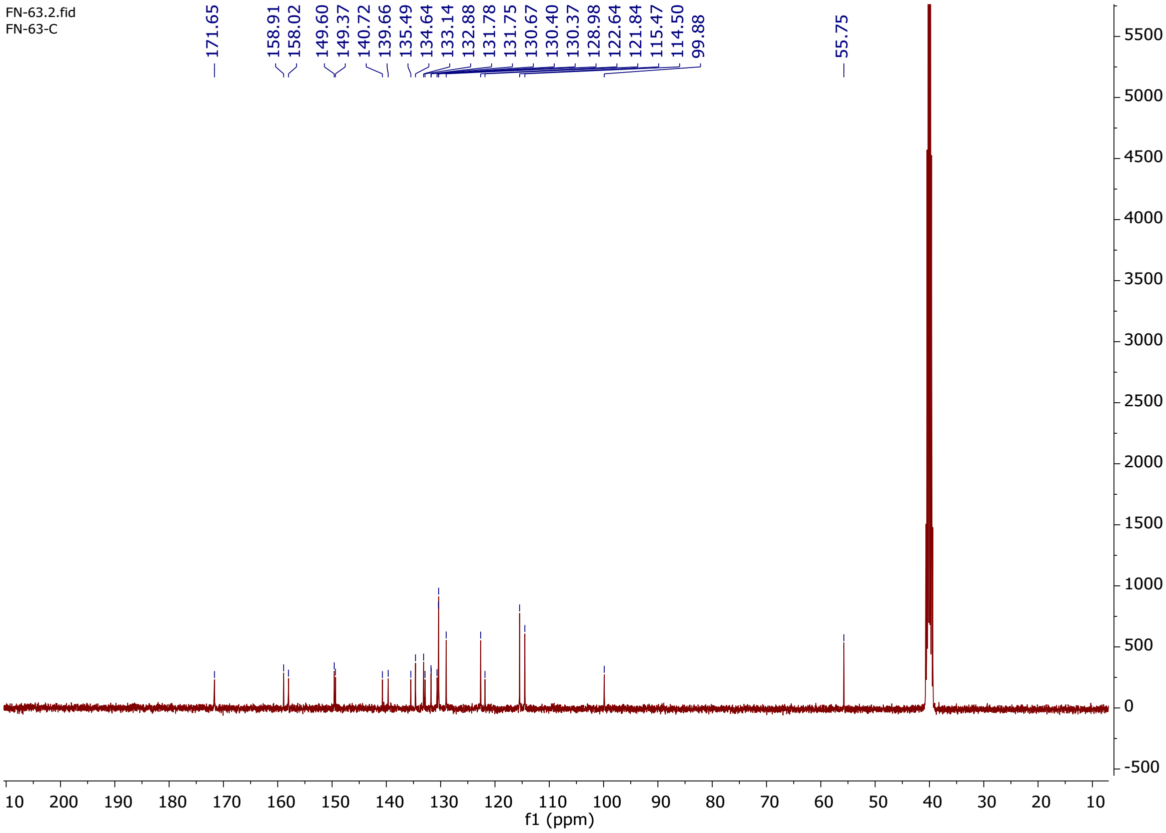

^{1}H and ^{13}C NMR of 3j

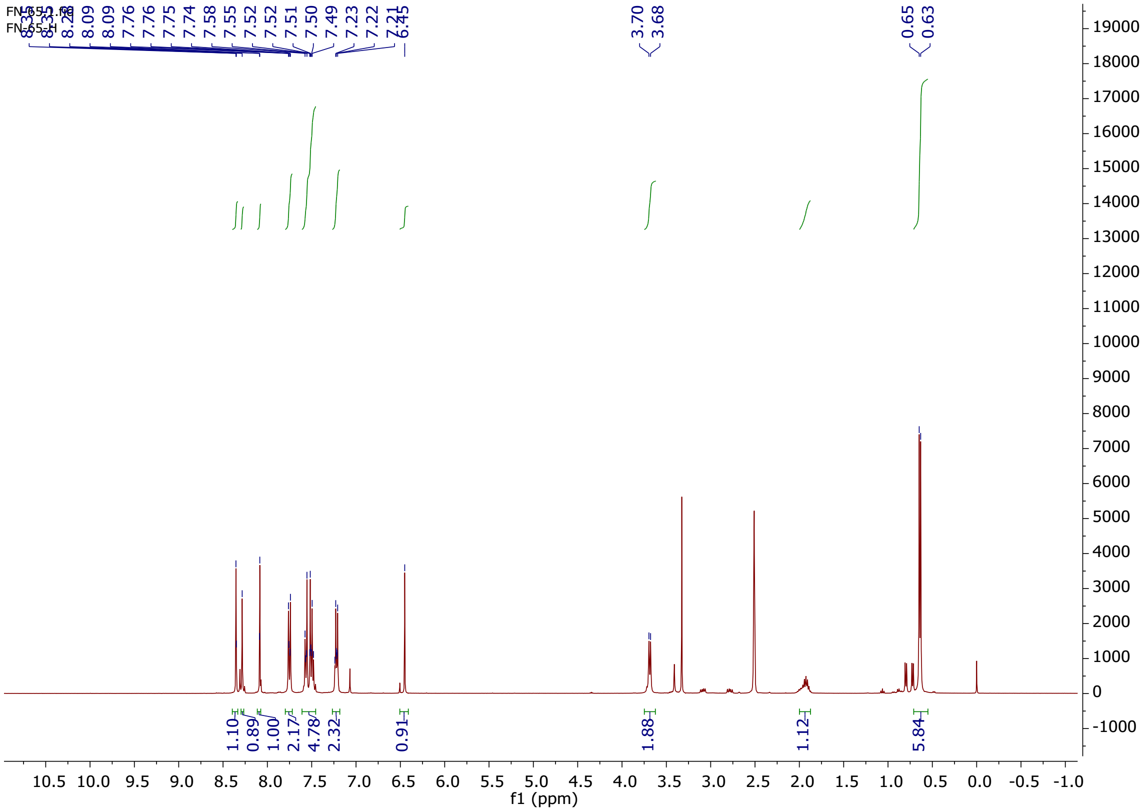

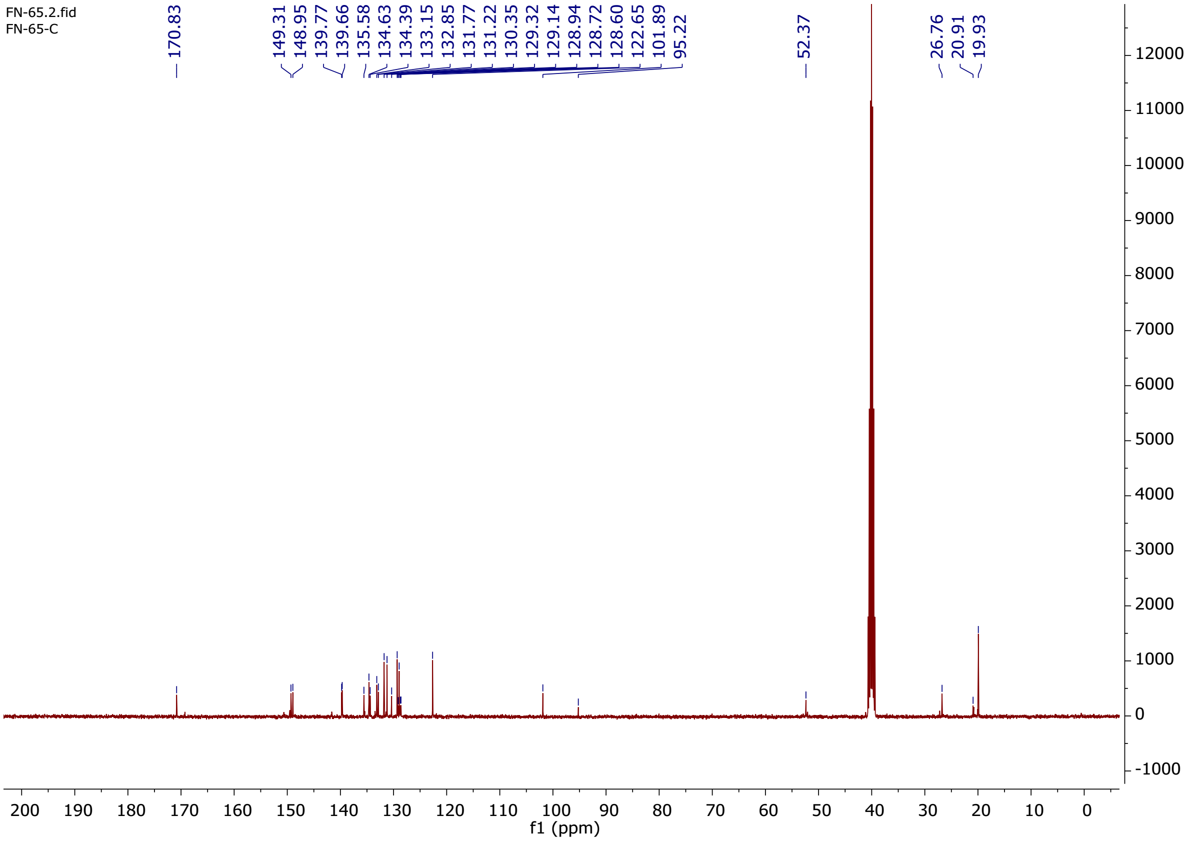

Supplement: RA-016-D6RA00305B-s001 [file RA-016-D6RA00305B-s001.pdf]
